# Unsupervised Learning‐Assisted Acoustic‐Driven Nano‐Lens Holography for the Ultrasensitive and Amplification‐Free Detection of Viable Bacteria

**DOI:** 10.1002/advs.202406912

**Published:** 2024-11-22

**Authors:** Yang Zhou, Junpeng Zhao, Junping Wen, Ziyan Wu, Yongzhen Dong, Yiping Chen

**Affiliations:** ^1^ State Key Laboratory of Marine Food Processing and Safety Control Dalian Polytechnic University Dalian Liaoning 116034 China; ^2^ Institute of Biopharmaceutical and Health Engineering Shenzhen International Graduate School Tsinghua University Shenzhen Guangdong 518055 China; ^3^ College of Engineering Huazhong Agricultural University Wuhan Hubei 430070 China; ^4^ College of Food Science and Technology Huazhong Agricultural University Wuhan Hubei 430070 China

**Keywords:** bacterial detection, CRISPR–Cas12a system, lens‐free holography, unsupervised learning

## Abstract

Bacterial infection is a crucial factor resulting in public health issues worldwide, often triggering epidemics and even fatalities. The accurate, rapid, and convenient detection of viable bacteria is an effective method for reducing infections and illness outbreaks. Here, an unsupervised learning–assisted and surface acoustic wave–interdigital transducer‐driven nano‐lens holography biosensing platform is developed for the ultrasensitive and amplification‐free detection of viable bacteria. The monitoring device integrated with the nano‐lens effect can achieve the holographic imaging of polystyrene microsphere probes in an ultra‐wide field of view (∽28.28 mm^2^), with a sensitivity limit of as low as 99 nm. A lightweight unsupervised learning hologram processing algorithm considerably reduces training time and computing hardware requirements, without requiring datasets with manual labels. By combining phage–mediated viable bacterial DNA extraction and enhanced CRISPR–Cas12a systems, this strategy successfully achieves the ultrasensitive detection of viable *Salmonella* in various real samples, demonstrating enhanced accuracy validated with the qPCR benchmark method. This approach has a low cost (∽$500) and is rapid (∽1 h) and highly sensitive (∽38 CFU mL^−1^), allowing for the amplification‐free detection of viable bacteria and emerging as a powerful tool for food safety inspection and clinical diagnosis.

## Graphical abstract

This study presents an unsupervised‐learning‐assisted, surface acoustic wave (SAW)–interdigital transducer (IDT)‐driven nano‐lens holography biosensing platform. By combining phage‐mediated viable bacterial DNA extraction and the enhanced CRISPR–Cas12a system, this approach demonstrates great potential for the sensitive and accurate quantification of viable pathogenic bacteria in complex sample matrices, offering a powerful tool for food safety inspection and clinical diagnosis.

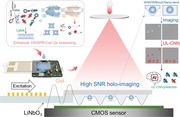

## 1 Introduction

As one of the important factors resulting in public health safety issues, bacterial infection often causes food poisoning, severe infections, and potential health risks, especially in resource‐poor developing areas.^[^ ^1^, ^2^ ^]^ The early diagnosis of bacteria requires a rapid, sensitive, and cost‐effective detection platform from the perspective of prevention, for the management of pathogen‐related accidents.^[^ ^3^, ^4^ ^]^ Real‐time quantitative polymerase chain reaction (qPCR) is extensively employed to detect bacteria and is considered a gold standard owing to its inherent merits, such as high sensitivity, exceptional specificity, and excellent reproducibility.^[^ ^5^, ^6^ ^]^ Furthermore, many emerging nucleic acid detection technologies offer many advantages due to their state‐of‐the‐art high‐sensitivity biosensing strategies and advanced data processing algorithms.^[^ ^7^, ^8^, ^9^, ^10^ ^]^ However, most of these strategies primarily depend on DNA amplification, require expensive fluorescence signal readout equipment, and are typically available only in specialized laboratories to avoid aerosol pollution.^[^ ^10^, ^11^ ^]^ Additionally, identifying viable bacteria has always been an insurmountable problem in traditional pathogen detection methods.^[^ ^12^, ^13^ ^]^ Hence, the development of sensitive and efficient biosensing strategies, along with the availability of affordable signal readout equipment, is crucial to the development of bacterial detection technologies.

With the development of highly integrated complementary metal oxide semiconductor (CMOS) image sensors, the small field of view (FOV) of traditional optical lens imaging can be offset with lens‐free holography.^[^ ^14^, ^15^ ^]^ Lens‐free holographic imaging can directly capture the hologram of the sample in the CMOS sensing area and digitally reconstruct the phase and amplitude of the target sample.^[^ ^16^, ^17^ ^]^ This approach typically offers a throughput considerably larger than the throughputs of traditional imaging devices. In our previous work,^[^ ^17^ ^]^ a multifunctional biosensing platform based on polystyrene (PS) microsphere single‐particle counting was successfully developed, employing a self‐constructed portable lens‐free holographic microscope for the sensitive detection of multitype targets. As ideal optical signal probes, functionalized PS microspheres offer excellent features, such as cost‐effectiveness, stable chemical properties, and uniform morphology.^[^ ^18^ ^]^ In general, small PS microsphere signal probes increase biochemical reaction efficiency, thereby improving detection sensitivity.^[^ ^19^, ^20^ ^]^ However, a portable holographic microscope is restricted by the poor signal‐to‐noise ratio (SNR) and cannot achieve ultra‐sensitive holographic imaging in an ultra‐wide FOV. Although lens‐free holographic imaging technologies can achieve ultra‐wide FOV digital imaging, its sensitivity is fundamentally constrained by factors, such as light diffraction and CMOS sensors, making high SNR holographic imaging a challenge.^[^ ^21^ ^]^ Holographic imaging has adopted a series of innovative strategies to tackle these challenges, including the virtual subdivision of each sensor pixel to enhance the SNR and the use of fluid polymers that form nano‐lenses, to boost contrast and signal levels from small nano‐particles.^[^ ^22^, ^23^, ^24^ ^]^ The high‐speed processing of large batches of holograms presents a considerable challenge to the realization of simultaneous ultra‐wide FOV and high SNR holographic imaging.

Deep learning–assisted technology has attracted considerable interest in recent years and has found widespread applications in various fields, including target detection, semantic segmentation, particle tracking, and classification.^[^ ^25^, ^26^, ^27^, ^28^ ^]^ Supervised learning, which is the most prevalent deep learning approach, utilizes high‐quality datasets containing substantial input data paired with corresponding expected outcomes to train deep neural networks and derive optimal models for solving specific problems.^[^ ^29^ ^]^ The acquisition and quality of a dataset are critical factors in the evaluation of training effectiveness. Publicly reported datasets rarely meet specific training requirements, and internally acquired datasets typically stem from experiments, demanding time‐consuming efforts and additional costs for annotating actual labels to achieve satisfactory training results.^[^ ^30^, ^31^, ^32^ ^]^ To break these restrictions, a novel deep‐learning method that can accomplish specific tasks by training on extremely limited datasets and eliminating the need for actual value labels should be developed.

**Scheme 1:**
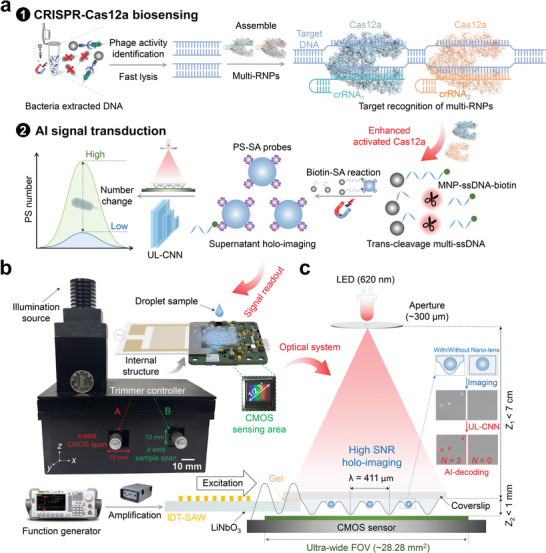
Schematic of UL‐SNH biosensing platform for bacterial detection. a) The biochemical reaction mechanism of UL‐SNH for bacterial detection, including CRISPR–Cas12a triggered biosensing and artificial intelligence (AI) signal transduction. b) SAW–IDT‐driven nano‐lens holography microscope. c) Optical system design of SNH microscope. The holographic imaging device of high SNR primarily consists of a CMOS sensor, a SAW–IDT chip, trimmer controllers, a coverslip, a function generator connected with amplification, and a partially coherent light source of modulated LED.

Herein, we developed an unsupervised‐learning (UL)–assisted and surface acoustic wave (SAW)–interdigital transducer (IDT)‐driven nano‐lens holography (UL‐SNH) biosensing platform for the ultrasensitive and amplification‐free detection of viable bacteria (**Scheme** **1**). In our strategy, phages specifically capture and enrich viable bacteria and induce them to release DNA, which is used as substrate DNA that activates the trans‐cleaving ability of clustered regularly interspaced short palindromic repeats (CRISPR)–CRISPR‐associated protein 12a (Cas12a) systems. These processes lead to the trans‐cleavage of biotin‐modified single‐stranded DNA (ssDNA). The ssDNA serves as a bridge to connect PS–streptavidin (SA) signal probes and magnetic nanoparticle (MNP)–ssDNA–biotin complexes through the bioorthogonal reaction between SA and biotin. Multiple target–specific CRISPR RNAs (crRNAs) can complex with Cas12a, forming ribonucleoproteins (RNPs) and thereby improving trans‐cleavage efficiency. Stepwise reagent addition triggers a biochemical reaction that changes the number of PS–SA probes in supernatants, and this process is directly related to the concentration of target bacteria. An SNH microscope was employed to capture the holograms of PS microsphere probes, and SNR was enhanced by manipulating a liquid to produce a localized nano‐lens effect, enabling ultra‐sensitive holographic imaging in an ultra‐wide FOV. UL–assisted image processing algorithms not only accomplish small‐batch training without requiring corresponding true value labels but also achieve accurate target detection. The multidisciplinary organic integration of the CRISPR–Cas12a system, SNH microscope, and UL–assisted image processing technology creates an innovative biosensing platform, offering a cost‐effective, user‐friendly, and field‐portable system for bacterial detection.

## 2 Results and Discussion

### 2.1 Principle and Design of the UL‐SNH Biosensing Platform

The UL‐SNH biosensing platform was mainly composed of three key parts: enhanced CRISPR–Cas12a biosensing, SNH microscope–based ultra‐sensitive holographic imaging with an ultra‐wide FOV, and UL–assisted image processing technology. As a proof of concept, the UL‐SNH biosensing platform was applied to detect bacteria, and a multiple crRNA–mediated CRISPR–Cas12a system was used (Scheme 1a). All DNA and RNA sequences are listed in Table S1 (Supporting Information). MNP–phages captured viable bacteria in various samples with high specificity to form MNP–phage–bacteria complexes, which were lysed rapidly to extract nucleic acid after magnetic separation enrichment. These crRNAs accurately recognized the specific DNA sequences of target bacteria and activated the trans‐cleavage activity of Cas12a enzymes. In our approach, PS–SA and MNP–ssDNA–biotin conjugates were combined to form PS–SA–biotin–ssDNA–MNP complexes. Cas12a enzyme complexes (Cas12a–multi‐crRNAs–target DNA) activated by target positive DNA trans‐cleaved ssDNA to prevent the formation of PS–MNP complexes. Non‐target negative DNA did not activate the cleavage activity of Cas12a enzymes to trans‐cleave ssDNA. After magnetic separation, PS–SA signal probes in the supernatant were collected for holographic imaging, and the convolutional neural network (CNN) completed signal transduction. The number of PS microsphere signal probes was directly related to the concentration of target viable bacteria without additional signal amplification. In other words, the high number of PS microspheres in a supernatant indicated the high concentration of bacteria.

An SNH microscope was assembled with a 3D‐printed physical shell and mainly composed of a CMOS sensor, a SAW–IDT chip, trimmer controllers, a coverslip, a function generator connected with amplification, and a partially coherent light source of modulated LED (Scheme 1b). Additional details of the SNH microscope construction can be found in the Supporting Information (Figure S1, Supporting Information). The SAW–IDT chip and CMOS imaging sensors were packaged inside as the key internal structure of the SNH microscope. The region of interest was obtained by adjusting the positions of these sensors with trimmer controllers. The CMOS sensor and SAW–IDT chip allowed for 15 and 10 mm span adjustments (accuracy: 0.01 mm) in the y‐ and x‐axes, respectively. To reduce detection costs and avoid cross‐contamination, the SAW–IDT chip can be reused and only needed to replace disposable coverslips coupled on the piezoelectric substrate surface. After approximately 600 times of reuse, the SAW–IDT chip electrodes showed slight wear, but it did not affect the overall performance (Figure S2, Supporting Information). The optical system design of the SNH microscope demonstrated the principle of SAW driving the liquid film to form nano‐lenses (Scheme 1c). The SAW–IDT chip using LiNbO_3_ as the piezoelectric substrate excited the Rayleigh wave at a design frequency of 9.71 MHz and then interacted with an ultrathin coverslip, generating a dispersive Lamb wave and inducing liquid deformation. Under the effect of SAW, the liquid thickness around the PS microspheres on the coverslip was changed to form sonic nano‐lenses and enhanced the SNR.^[^ ^23^ ^]^ Our strategy did not break through the pixel size limit of CMOS sensors but enhanced the holographic imaging sensitivity through a new optical and acoustic method.

It is worth noting that light intensity and working distances are crucial to the performance of holographic imaging. The SNH microscope utilized a modulated LED (620 nm) as the partially coherent light source, with its intensity adjustable via an integrated microcontroller (Figure S3a, Supporting Information). Additionally, the CMOS image sensor (IDS UI‐3592LE‐C) in our system, along with the supporting driver software (IDS uEye Cockpit), enabled adaptive contrast adjustments to further minimize the impact of light intensity (Figure S3b, Supporting Information). For working distances, two parameters are crucial for holographic imaging: the distance *Z*_1_ from the light source to the sample and *Z*_2_ from the sample to the CMOS imaging area. In our strategy, 3D‐printed detachable coarse adjustment modules of different heights (0.5, 1, and 2 cm; Figure S3c, Supporting Information), combined with the fine adjustment module of the micro‐displacement stage (Figure S3d, Supporting Information), were employed to adjust the *Z*_1_ distance. A disposable coverslip (∽120 µm) was placed over the CMOS sensor area to ensure that the *Z*_2_ < 1 mm. According to the design parameters of the SNH microscope, the working distances of *Z*_1_ and *Z*_2_ were optimized to ∽6–6.5 cm and < 1 mm (*Z*_1_/*Z*_2_ ≫ 1), respectively. After capturing high SNR holograms, UL–assisted image processing technology processed these images with a rapidly trained CNN and marked the identified PS microspheres, and the output was used in the calculation of the target bacterial concentration. Owing to the organic combination of interdisciplinary fields, efficient biochemical reactions, and advanced image processing technology, the UL‐SNH emerges as a universal detection platform that can be easily integrated into other biological detection strategies for point‐of‐care testing.

### 2.2 Finite Element Simulation and Numerical Analysis

To verify the feasibility of generating the SAW waves with the SAW–IDT resonator, we conducted a multi‐physics coupling simulation analysis of the piezoelectric effect at a characteristic frequency. Given that the simplification of models can effectively reduce the calculation time, the SAW–IDT model was equivalently simplified before the simulation analysis. A schematic of the simplified SAW–IDT resonator is shown in Figure S4a (Supporting Information), and a pair of the SAW–IDT resonators in propagation is cut to a cross section to transform into an equivalent 2D geometry. When the input excitation frequency matches the designed resonant frequency of the SAW–IDT chip, the LiNbO_3_ substrate can generate potential differences (Figure S4b, Supporting Information) and corresponding electric field modulus, causing a piezoelectric effect (Figure S4c, Supporting Information). This piezoelectric effect can convert potential difference into mechanical energy, thereby deforming the piezoelectric substrate and generating Rayleigh waves propagating forward on the substrate. Figure S4d (Supporting Information) shows the displacement vibration changes in the piezoelectric substrate in the Rayleigh wave antisymmetric mode caused by the piezoelectric effect. Notably, the mechanical wave intensity generated by the SAW–IDT resonator is a linear superposition of each pair of interdigitated electrodes and is positively related to the number of pairs of electrodes. The successful generation of SAW was accompanied by energy transfer that changes the thickness of a thin liquid layer, subsequently influencing the local optical amplification effect of PS microspheres.

To explore the PS microsphere particle trajectory in the liquid layer under the acoustic radiation force, we developed a simplified liquid–glass 2D model for fluid–solid coupling simulation (Figure S4e, Supporting Information). As the thickness of the liquid layer increased and exceeded the particle size of the PS microspheres, the Lamb wave formed by the coupling of the mechanical wave propagating on the piezoelectric substrate to the cover glass leaked into the sample, resulting in the generation of acoustic radiation force. The simulation results demonstrated that the PS microspheres occur in relative motion, which was primarily controlled by the acoustic radiation force that concentrates the particles on the pressure node (Figure S4f, Supporting Information). As the thickness of the liquid layer increased, the Lamb waves that leaked into the liquid layer increased, leading to acoustic convection. This phenomenon induced the movement of the PS microspheres, which exerted a detrimental effect on the formation of nano‐lenses. Hence, the liquid film layer should be as thin as possible to minimize Lamb wave leakage, which is the key to forming nano‐lenses.

Additionally, as illustrated in Figure S4g (Supporting Information), when SAW propagates within a thin plate, the phase velocity changes at an excitation frequency range of 0–60 MHz, and different excitation frequencies can trigger distinct propagation modes, including symmetric and antisymmetric modes (S and A modes). For specific requirements, high excitation frequencies can initiate higher‐order modes (S1, A1, S2, A2, S3, and A3 modes), increasing the number of oscillation cycles and nodes and enhancing performance. Simulation and numerical analysis results indicated that the SNH microscope can generate SAW dominated by the A0 mode at a design frequency of 9.71 MHz, transferring energy to the thin liquid layer of disposable coverslips and controlling its thickness to produce nano‐lenses. Different from the instantaneous high SNR strategy, the UL‐SNH biosensing platform requires the generation of a large number of stable and long‐lasting nano‐lens for accurate detection. When the excitation signal persisted at a high frequency and energy transfer rate, interface loss inevitably increased during SAW coupling propagation, leading to thermal effects in the liquid medium. This thermal effect induced the evaporation of the liquid layer on the surface of the disposable coverslip, decreasing the thickness of the liquid layer and stopping the relative motion of the PS microspheres. Consequently, nano‐lens formed at the PS microsphere probe, and the formation was not limited to the antinode position of the SAW. The thermal energy generated by the CMOS imaging chip further accelerated this phenomenon.

### 2.3 UL–Assisted Image Processing and Sensitivity Evaluation

**Figure 1:**
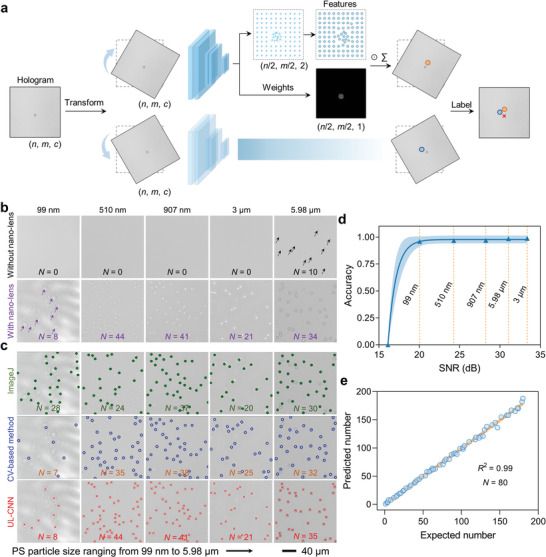
Evaluation of UL–assisted image processing method for detecting different PS microsphere holograms captured from the SNH microscope. a) Schematic of UL‐CNN method to detect single PS microsphere hologram. b) Evaluation of SNH microscope holographic imaging sensitivity limit of PS microsphere with different particle sizes (99 nm, 510 nm, 907 nm, 3 µm, and 5.98 µm). c) Comparison of counting results between UL‐CNN and representative image processing methods, including ImageJ and CV–based methods. d) Detection accuracy of UL‐CNN on different PS particle sizes. e) Correlation analysis between the expected (manual counting) and predicted number (UL‐CNN counting) of different PS particle sizes (99 nm, 510 nm, 907 nm, 3 µm, and 5.98 µm) detected by the UL‐CNN method (*N* = 80 images).

The UL–assisted image processing method is a label‐free target detection algorithm based on geometric deep learning (e.g., UL‐CNN method), which can predict the location of detection targets through a series of geometric transformations, including translations, reflections, and rotations. Traditional target detection algorithms, such as You Only Look Once (YOLO) and the Fast Region–based CNN, often struggle to achieve accurate target detection without access to real label values.^[^ ^17^, ^33^ ^]^ Our strategy identifies target centers by leveraging the symmetry axis in geometric transformations,^[^ ^34^ ^]^ enabling the UL‐CNN to accurately locate a target. Here, we demonstrated that UL‐CNN detected a single PS microsphere in the hologram as an example to exhibit the process of locating the particle position without ground labels (**Figure** **1**a). The transformed microsphere image (*n* × *m* pixels, *c* color channels) was input to UL‐CNN comprising a convolutional layer, max pooling layer, and additional convolutional layer for the prediction of microsphere position. For each transformed image, UL‐CNN generated two feature tensors: a vector field (*n*/2 × *m*/2 pixels, two color channels) and a weight map (*n*/2 × *m*/2 pixels, one color channel). The vector field represented the direction and distance from the pixel itself to the object, and the weight map indicated the contribution of each element to the final prediction. The predicted value of a single microsphere position after a transformation was calculated by multiplying and summing the two feature tensors and averaging the detected object position through a weight map. To ascertain the ultimate position of the detected PS microsphere, an inverse transformation was applied to convert the predicted position images back to their original form. The UL‐CNN enabled small‐batch training with a single‐feature microsphere image, and the entire training process was completed on a low‐configuration computer within a few minutes due to the small size of the deep neural network and the absence of complex graphics operations.

Before the accuracy of UL‐CNN in detecting different PS microsphere holographic images was evaluated, the sensitivity limitation of the SNH microscope was assessed using PS microspheres with different particle sizes (99 nm, 510 nm, 907 nm, 3 µm, and 5.98 µm). Observing PS microspheres with particle sizes smaller than 6 µm with the SNH microscope was difficult without the aid of the nano‐lens effect (Figure 1b). Owing to the low SNR of the optical system, 6 µm PS microspheres were directly observed, and the target signal overlapped with background noise, posing challenges to the extraction of PS microsphere image information. Nevertheless, the sensitivity limitation of the SNH microscope improved to 99 nm when PS microspheres formed stable nano‐lenses. In addition, this stable nano‐lens structure, generated by the SAW manipulation of the thickness of a liquid layer, can be maintained at room temperature for over 10 min without the requirement for continuous SAW excitation. In micron‐scale PS microsphere ultra‐sensitive holographic imaging, the nano‐lens effect is similar to the focusing capability of traditional optical microscopes, yielding a high SNR in the acquired holographic images, which can be directly processed using the UL‐CNN for target detection without extra holographic reconstruction. Although the SNH microscope allowed for the clear observation of the holographic images of PS microspheres with different particle sizes, the imaging SNR of nanoscale microspheres still needs to be improved with our strategy.

Compared with conventional optical microscope imaging and direct holographic imaging without the nano‐lens effect in our previous work,^[^ ^17^, ^20^ ^]^ our approach demonstrated nearly 10‐ (99 nm vs 1 µm) and 101‐fold (99 nm vs 10 µm) improvement in sensitivity, respectively (Table S2, Supporting Information). The nano‐lens effect was employed to enhance contrast and signal levels from small PS microsphere probes, allowing them to be separated easily relative to background noise without complex digital holographic reconstruction. While ensuring analytical performance, the computational effort required for hologram processing was significantly reduced, particularly when processing a large number of holograms simultaneously. Further, the USAF 1951 resolution test chart was employed for imaging with the SNH microscope and a conventional optical microscope with different magnification objective lenses for the comparison of FOVs. The results showed that the SNH microscope had an ultra‐wide FOV of 28.275 mm^2^, which was nearly 7‐, 39‐, and 155‐fold the FOV of the optical microscope: 4 × (FN20, 0.1; 3.990 mm^2^), 10 × (FN25, 0.25; 0.723 mm^2^), and 20 × (FN25, 0.4; 0.182 mm^2^) objective lenses, respectively (Figure S5, Supporting Information). The larger FOV and higher sensitivity of the SNH microscope allowed for the observation of a greater number of smaller‐sized PS microspheres relative to the number that can be observed with other lens–based imaging devices. This feature is essential for the detection accuracy of biosensing platforms.

To assess the accuracy of a UL–assisted image processing method in detecting different PS microspheres captured by the SNH microscope, we used the five representative images of different particle sizes (99 nm, 510 nm, 907 nm, 3 µm, and 5.98 µm; 512 × 512 pixels) and compared them by using three distinct image processing approaches: ImageJ,^[^ ^35^ ^]^ computer vision (CV)–based,^[^ ^36^ ^]^ and UL‐CNN methods. All PS microspheres identified through various methods were marked with different colors, and the counting results were then compared with real values (Figure 1c). The counting results showed that neither the ImageJ nor CV–based method was able to extract PS microsphere features in the 99 nm hologram with a low SNR for accurate identification. Notably, during the identification of holograms of different particle sizes with a high SNR, ImageJ and CV–based methods encountered varying degrees of error or feature loss. By contrast, the UL‐CNN method can accurately identify and mark these PS microspheres in holograms of different particle sizes and SNR, with an accuracy exceeding 98% (Figure 1d).

Given the efficiency of biochemical reactions, SNRs of these holograms, and the number of background microspheres, 3 µm PS microspheres with the highest SNR were finally selected in the next step. The background number and biochemical reaction efficiency were significantly improved (*P* < 0.0001, *α* = 0.05, unpaired two‐tailed Student's *t*‐test) when small‐size PS microspheres were employed as signal probes (Figure S6, Supporting Information). Notably, the SNH microscope captured the nano‐lens holograms of the 3 µm pure PS microspheres with high sensitivity and good reproducibility, demonstrating an excellent linear relationship between various concentrations and particle numbers with *R* ^2^ of 0.99 (Figure S7, Supporting Information). In addition, holograms of different particle sizes generated during the experiment were analyzed using UL‐CNN, and the predicted number (UL‐CNN counting) of the PS microspheres was compared with the expected number (manual counting). The analysis results of 80 images showed that the predicted number and expected number had a good linear correlation with *R* ^2^ = 0.99 and ranged from 0 to 200 (Figure 1e). These holograms were obtained from pure PS microspheres at different concentrations (without biochemical reactions involved) or generated during experiments. Compared with the YOLOv7–based supervised algorithm in our previous work,^[^ ^17^ ^]^ the lightweight UL‐CNN architecture reduced training time, training epochs, and GPU bandwidth requirement by nearly 70‐ (3 min vs 3.5 h), 8.3‐ (30 vs 250 epochs), and 7.9‐fold (128 vs 1008 GB/s; Table S3, Supporting Information). Owing to the lightweight framework, the UL‐CNN easily handled PS microsphere holograms with particle sizes ranging from 99 nm to 5.98 µm without the need for the complex training of human‐marked labels. This high‐speed and high‐throughput image processing method considerably improved detection efficiency and minimized human labeling errors, overcoming the traditional algorithm requirement of large datasets for training and enhancing the versatility and precision of the UL‐SNH biosensing platform.

### 2.4 Feasibility and Characterization of UL‐SNH Biosensing Platform

**Figure 2:**
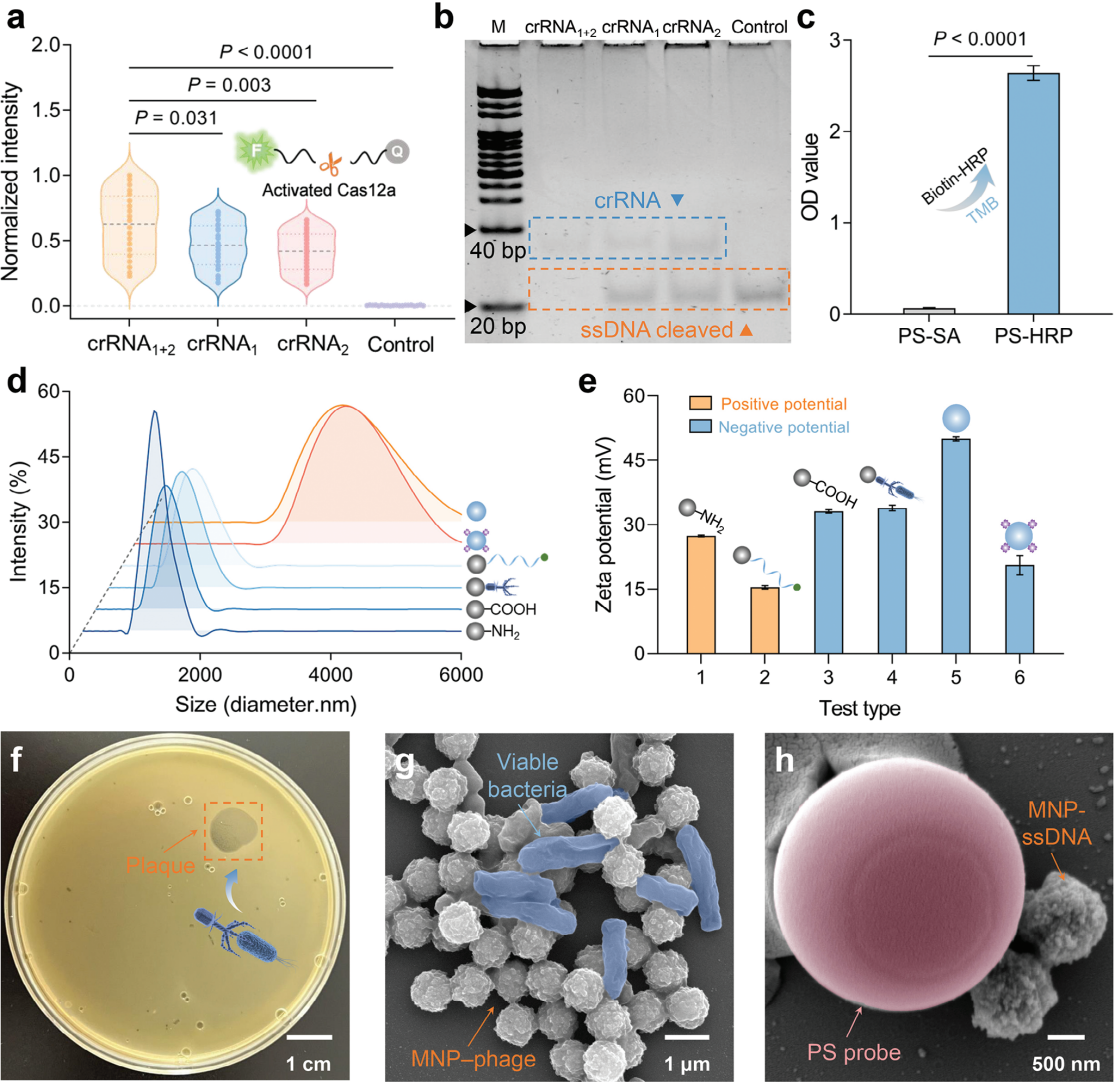
Feasibility and characterization of UL‐SNH biosensing platform. a) Fluorescence intensity generated by different crRNA combinations activating Cas12a to cleave FAM probes. b) Different crRNA combinations activating Cas12a to cleave ssDNA probes were verified by polyacrylamide gel electrophoresis. “M” represents 20 bp DNA Ladder. c) Color development of PS–SA–biotin–HRP in the TMB substrate. d) Change in the hydrodynamic size distribution of PS microsphere and MNP conjugates. e) Change in zeta potential of PS microsphere and MNP conjugates. f) Viable *S. typhimurium* infected by LPST10 phages form plaques on a plate. SEM images of g) viable *S. typhimurium* captured by MNP–LPST10 phage and h) PS–ssDNA–MNP complexes. *n* = 3 technical replicates, unpaired two‐tailed Student's *t*‐test, error bars represent mean ± standard deviation (SD).

For conventional CRISPR–Cas12a–mediated bacterial detection methods, appropriate DNA amplification can greatly enhance the trans‐cleavage efficiency of Cas12a and reduce the detection time, but it increases detection costs and the risk of aerosol contamination. To evaluate the trans‐cleavage ability and feasibility of the enhanced activated Cas12a without amplification, we activated various crRNA combinations (crRNA_1_, crRNA_2_, and crRNA_1+2_) assembled with Cas12a produced by *S. typhimurium* DNA to cleave the 5–carboxyfluorescein (FAM) probes and ssDNA. The unpaired two‐tailed Student's *t*‐test results revealed significant statistical differences (*P* = 0.031 and 0.003) at *α* = 0.05 in the normalized fluorescence intensity produced by different crRNA combinations activating Cas12a and cleaving the FAM probes (**Figure** **2**a). Multiple crRNAs can assemble Cas12a and form additional RNPs, thereby enhancing Cas12a trans‐cleaving activity and cleaving FAM probes. The trans‐cleaving ability of Cas12a can only be activated in the presence of specific crRNAs (*P* < 0.0001). Additionally, polyacrylamide gel electrophoresis (14%) results showed that the combination of multiple crRNAs exerted positive effects on the cleavage efficiency of the ssDNA probes (Figure 2b). The band corresponding to ssDNA disappeared after it was cleaved multiple times by Cas12a activated with crRNA_1_ and crRNA_2_. By contrast, Cas12a activated by a single type of crRNA only partially cleaved the ssDNA probe, resulting in light bands. As a DNA molecular weight reference, the 20 bp band of the DNA ladder indicated the positions of crRNAs (44 nt) and ssDNA probes (25 nt). The cleavage results of FAM and ssDNA probes showed that Cas12a activated by the multi‐crRNA assembly had satisfactory cleavage efficiency, thus proving the feasibility of the UL‐SNH biochemical reaction strategy.

To verify the success of the PS microspheres and MNPs coupling strategy, the conjugates were characterized in terms of color reaction, dynamic light scattering, and zeta potential. Horseradish peroxidase (HRP) can catalyze 3,3′,5,5′–tetramethylbenzidine (TMB) substrate to produce a blue change visible to the naked eye. Optical density (OD) results indicated that a biotin‐labeled HRP was effectively bound with the PS–SA conjugates, forming PS–SA–biotin–HRP complexes that catalyzed the formation of colorless TMB and changed the color to blue (Figure 2c). The unpaired two‐tailed Student's *t*‐test results statistical difference in OD values (*P* < 0.0001) at *α* = 0.05 before and after color development, indicating the successful preparation of the PS–SA conjugates. In addition, dynamic light scattering particle size analysis results showed that the initial hydrodynamic sizes of the MNPs and PS microspheres were approximately 1032 and 3090 nm, respectively (Figure 2d). Subsequent coupling with various types of biorecognition molecules induced changes in the hydrodynamic size and intensity of the MNPs and PS microspheres, thereby confirming the success of coupling strategies. The absolute values of the zeta potential were greater than 15 mV, indicating that the dispersion of these conjugates had good stability (Figure 2e). To demonstrate the specificity and feasibility of phages in capturing viable bacteria, LPST10 phages were employed to infect *S. typhimurium* on the plate. Plaques became clearly visible on the plate, indicating that the LPST10 phages specifically infected viable *S. typhimurium* and naturally lysed it (Figure 2f). To further confirm that viable bacteria were captured by LPST10 phages and the reaction between SA and biotin occurred, we characterized the MNP–phage–bacteria and PS–SA–biotin–ssDNA–MNP complexes with a scanning electron microscope (SEM). The MNP–phage conjugates successfully captured the viable *S. typhimurium*, which was used for rapid nucleic acid extraction after magnetic separation (Figure 2g). Furthermore, the SEM image provided additional evidence that the MNPs were successfully bound to the surfaces of the PS microspheres after the SA–biotin reaction, and the particle number in the supernatant changed after magnetic separation (Figure 2h). Hence, these experimental findings and characterization results validated the feasibility of the UL‐SNH biosensing platform, establishing a foundation for the accurate detection of bacteria.

### 2.5 UL‐SNH Biosensing Platform for *S. typhimurium* Detection

**Figure 3:**
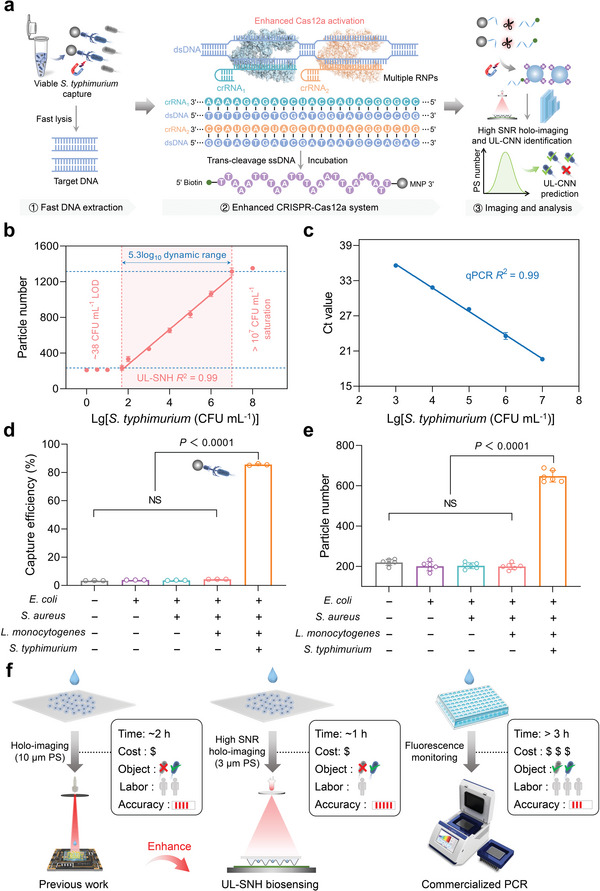
*S. typhimurium* detection results of UL‐SNH biosensing platform. a) Schematic and workflow of UL‐SNH biosensing platform for *S. typhimurium* detection. b) Standard curve and linear range of UL‐SNH biosensing platform for *S. typhimurium* detection. c) Linear range of qPCR method for *S. typhimurium* detection. Specificity analysis of UL‐SNH biosensing platform for d) LPST10 phage–mediated viable bacterial DNA extraction (*n *= 3 technical replicates) and e) *S. typhimurium* detection in various bacterial interference (*n *= 6 technical replicates), including *E. coli*, *S. aureus*, and *L. monocytogenes*. f) Performance comparison of representative diagnostic platforms (previous work, UL‐SNH, and commercialized PCR method) for *S. typhimurium* detection. “+” and “−” represent positive and negative, respectively. “NS” represents no significance with *p* > 0.05. Unpaired two‐tailed Student's *t*‐test, error bars represent mean ± SD.

Among the various types of bacterial food poisoning reported in countries worldwide, *Salmonella* consistently emerges as one of the most hazardous bacteria, frequently topping the list.^[^ ^37^ ^]^ *S. typhimurium* exhibits higher specificity for human hosts than other *Salmonella* subspecies, resulting in severe infections that can lead to death.^[^ ^38^ ^]^ **Figure** **3**a shows the schematic and workflow of the UL‐SNH biosensing platform for *S. typhimurium* detection. Before UL‐SNH was used to detect *S. typhimurium*, most essential parameters affecting the biosensing platform accuracy were systematically optimized, including phage–mediated viable bacterial DNA extraction, nano‐lens formation, and CRISPR–Cas12a system. For DNA extraction, MNP particle size, EDC/NHS concentration, LPST10 phage dosage, and MNP–phage coupling time were optimized for the efficient coupling of MNPs and LPST10 phages, and the optimal values were 1000 nm, 10 mg mL^−1^, 40 µL, and 3 h (Figure S8, Supporting Information). In addition, the optimized MNP–phage concentration, the volume ratio of *S. typhimurium* to MNP–phage, and capture time were 500 µg mL^−1^, 1:3, and 15 min, respectively (Figure S9, Supporting Information). Under the above optimal conditions, the MNP–phage–mediated capture efficiency of viable *S. typhimurium* reached nearly 85%. Subsequently, we optimized key conditions for the nano‐lens formation of 3 µm PS microspheres and the number of PS–ssDNA–MNP complexes. These conditions were optimized in terms of PEG–600 ratio and MNP–ssDNA concentration to control the thickness of the liquid layer and calibrate the number of background PS microspheres. The optimal conditions of the PEG–600 ratio and MNP–ssDNA concentration were 50% and 80 µg mL^−1^, respectively (Figure S10, Supporting Information). Under optimal conditions, the number of 3 µm PS microspheres forming nano‐lenses reached saturation, whereas background PS microspheres after the bioorthogonal reaction exhibited minimal interference and remained stable. Furthermore, we systematically optimized the key parameters of the enhanced CRISPR–Cas12a system, including the ratio of Cas12a to crRNAs, Cas12a concentration, and cleavage time to 1:2, 1 µm, and 60 min, respectively, to ensure the accuracy of CRISPR biosensing (Figure S11, Supporting Information). The optimization of all conditions and calibration of the number of PS microspheres established the foundation for the quantitative detection of *S. typhimurium* on the UL‐SNH biosensing platform.

Under optimal conditions, the sensitivity of the UL‐SNH biosensing platform for bacterial detection was evaluated using the gradient dilutions of *S. typhimurium* at various concentrations ranging from 1 × 10^0^ to 1 × 10^8^ CFU mL^−1^. The nano‐lenses of PS microspheres observed in the supernatant using an SNH microscope exhibited a positive correlation with the concentration of *S. typhimurium* (Figure S12, Supporting Information). In addition, a good linear relationship was indicated between the number of PS microspheres and *S. typhimurium* concentration ranging from 50 × 10^0^ to 1 × 10^7^ CFU mL^−1^, and the linear equation was *Y* = 196.9 *X* – 109.3, *X* = Lg [*S. typhimurium* (CFU mL^−1^)], *R* ^2^ = 0.99 (Figure 3b). The limit of detection (LOD) for *S. typhimurium* was ∽38 CFU mL^−1^ (*S* = 10.66, *M* = 0.85) according to the formula LOD = 3*S*/*M*, where *S* is the value of the standard deviation of blank samples and *M* is the slope of the standard curve at a low concentration range. In addition, the qPCR method was employed as a benchmark for the detection of *S. typhimurium*. A good linear relationship was demonstrated between the cycle threshold (Ct) and the *S. typhimurium* concentration ranging from 1 × 10^3^ to 1 × 10^7^ CFU mL^−1^ (*R* ^2^ = 0.99; Figure 3c). The UL‐SNH enabled the easy enrichment and amplification‐free detection of viable bacteria at room temperature. This feature is challenging to achieve with the qPCR method.

The selectivity of the UL‐SNH biosensing platform primarily relies on the double guarantee of LPST10 phages and the CRISPR–Cas12a system. To evaluate the specificity of the UL‐SNH biosensing platform, we used various species of bacteria, including *Escherichia coli* (*E. coli*), *Staphylococcus aureus* (*S. aureus*), and *Listeria monocytogenes* (*L. monocytogenes*), as interference matrices to verify phage–mediated DNA extraction and CRISPR biosensing. The unpaired two‐tailed Student's *t*‐test results indicated that the LPST10 phages exhibited high specificity and satisfactory capture efficiency for *S. typhimurium* (*P* < 0.0001; *α* = 0.05) while showing minimal effect against other interfering bacteria (Figure 3d). The high specificity of phage infections was attributed to their recognition mechanism, and they targeted specific receptor molecular structures, such as proteins or polysaccharide structures, on the bacterial surface. In addition, the unpaired two‐tailed Student's *t*‐test results of UL‐SNH for *S. typhimurium* detection indicated that only *S. typhimurium* DNA activated Cas12a's trans‐cleavage activity and resulted in changes in the number of PS microspheres in the supernatant (*P* < 0.0001; *α* = 0.05). Other interfering DNA did not cause considerable changes (Figure 3e). This phenomenon occurred because Cas12 was activated and cleaved ssDNA probes only when the crRNA completely matched the conserved DNA sequences released from viable *S. typhimurium*. Therefore, the UL‐SNH biosensing platform exhibited excellent specificity under a double guarantee. Furthermore, UL‐SNH was employed to detect various concentrations of *S. typhimurium* spiked into pork samples (1 × 10^3^, 1 × 10^4^, and 1 × 10^5^ CFU mL^−1^), which were pretreated according to the Chinese national standard GB 4789.4–2016. The results showed that the spiked recovery rates of the samples were 94.9–109.3%, and the coefficient of variation was less than 13.0%, proving that the UL‐SNH biosensing platform had good accuracy and reproducibility (Table S4, Supporting Information).

The UL‐SNH typically provided a cost‐effective, user‐friendly, and accurate biosensor platform, integrating with phage–mediated DNA extraction, UL–assisted image processing algorithm, enhanced CRISPR–Cas12a system, and SNH microscope with ultra‐wide FOV. Compared with the method proposed in our previous work^[^ ^17^ ^]^ and the commercialized PCR method, the UL‐SNH offered advantages for bacterial detection and exhibited superior performance in terms of time (∽1 h), cost (≈∽$500), object (viable bacteria), labor (easy operation), and accuracy (∽38 CFU mL^−1^; Figure 3f; and Table S5, Supporting Information). The detection sensitivity of UL‐SNH increased two‐fold, and the detection time was shortened by 30 min (Figure S13, Supporting Information). Furthermore, our strategy shortened the detection time by nearly three‐fold relative to a commercial PCR method and identified viable bacteria without pre‐amplification. Compared with other bacterial detection methods, including amplification and amplification‐free methods, UL‐SNH offered many advantages in terms of sensitivity, linear range, DNA extraction, detection time, overall costs, and signal readout strategy (Table S6, Supporting Information). Additionally, our strategy also demonstrated significant performance improvements over similar studies that applied only one or two technologies, effectively overcoming challenges inherent in their respective fields (Table S7, Supporting Information). However, it should be noted that some bacteria may carry relevant genes without expressing them, making them not pathogenic. Therefore, DNA‐based bacterial detection methods mainly indicate potential pathogenic risks associated with bacteria, which is also essential for related disease prevention.

### 2.6 Real Sample Analysis

To assess the analytical capabilities of the UL‐SNH biosensing platform in real‐world applications, we employed different categories of real samples for *S. typhimurium* detection, including artificially contaminated food samples (*N *= 45) and clinical samples (*N *= 15).

**Figure 4:**
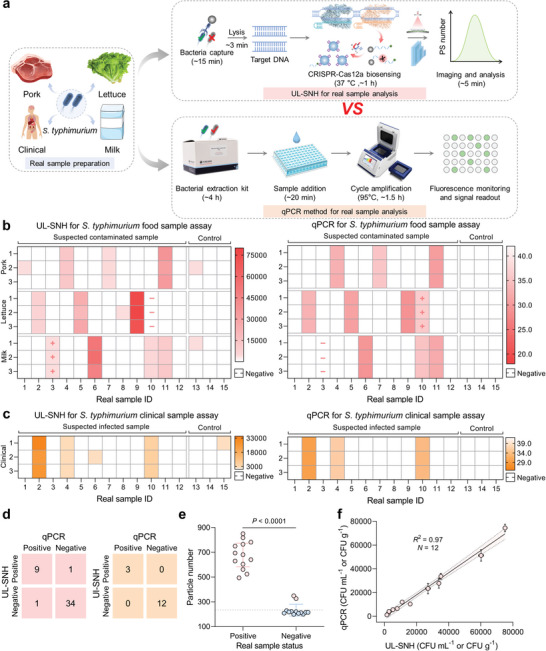
Real sample analysis of UL‐SNH biosensing platform for *S. typhimurium* detection. a) Schematic of UL‐SNH and qPCR method for real sample analysis. b) Artificially contaminated food samples and c) clinical samples assayed by UL‐SNH and confirmed by standard qPCR method. The heatmap represents the *S. typhimurium* concentrations (left panel) and Ct values (right panel) of each sample. “−” and “+” represent negative and positive detection results, respectively. d) Confusion matrix summarizing the detection performance between positive and negative samples of food samples and clinical samples by UL‐SNH and qPCR method. e) Difference analysis between positive and negative real sample status by UL‐SNH (*N* = 26 samples). f) Correlation analysis of UL‐SNH and qPCR detection results in positive real samples (*N* = 12 samples). *n* = 3 technical replicates, unpaired two‐tailed Student's *t*‐test, error bars represent mean ± SD.

All the samples were divided into experimental groups (*N *= 48, Nos. 1–12) and control groups (*N *= 12, Nos. 13–15) in a ratio of 4:1 and pretreated according to the Chinese national standard (GB 4789.4–2016). Detailed preparation processes for the artificially contaminated food samples and the clinical samples were provided in the Supporting Information. These artificially contaminated food samples contained a low‐concentration *S. typhimurium* sample that exceeded the LOD of qPCR and a non‐viable *S. typhimurium* sample. These clinical samples were collected from patients during microbiological examinations, with sample pre‐treatment and plate screening culture conducted at the General Hospital of Central Theater Command (Wuhan, China, 2023‐026‐01). For the analysis of these samples, the qPCR method of the Chinese industry standard (SN/T 1870–2016) was employed as the benchmark for investigating the accuracy of our strategy (**Figure** **4**a). The heatmap showed the *S. typhimurium* detection results of the UL‐SNH and qPCR methods for different artificially contaminated food and clinical samples (Figure 4b,c). The confusion matrix summarized the positive and negative detection results of *S. typhimurium* obtained by the two methods (Figure 4d). UL‐SNH and qPCR methods detected 9 positive food samples (Nos. 4, 7, and 11 of pork samples; Nos. 2, 5, and 9 of lettuce samples; Nos. 6, 10, and 11 of milk samples), 34 negative food samples, 3 positive clinical samples (Nos. 2, 4, and 10), and 12 negative clinical samples. Owing to the high sensitivity of the UL‐SNH biosensing platform, the low‐concentration *S. typhimurium* contamination sample (Nos. 3 of the milk sample) was detected positive by UL‐SNH but was not accurately identified by the qPCR method. Additionally, UL‐SNH successfully identified the live bacteria in artificially contaminated food samples (No. 10 of the lettuce sample) and had no false positives. The second feature is crucial for practical applications in bacterial detection. By contrast, qPCR failed to accurately detect the No. 10 lettuce sample. Although UL‐SNH might yield some false positives when assaying negative samples, the final results were consistent with the benchmark. The unpaired two‐tailed Student's *t*‐test results indicated a statistical difference in the number of PS microspheres between positive and negative samples (*P* < 0.0001, *α* = 0.05; Figure 4e). Furthermore, the correlation analysis results demonstrated good consistency between the concentration of *S. typhimurium* in the positive real samples detected by UL‐SNH and qPCR methods, with *R* ^2^ of 0.97 (Figure 4f). Overall, UL‐SNH holds great potential for food safety inspection and the rapid clinical diagnosis of bacterial infection.

Although UL‐SNH has achieved the ultra‐sensitive detection of *S. typhimurium* in a variety of real samples, this work still has several limitations that need further improvement. The sample preparations and processing steps need to be further automated and simplified, which is crucial for on‐site detection. The development of automated pre‐treatment devices, combined with standardized processing steps, can significantly enhance efficiency to meet the requirements for complex sample detection. Additionally, further exploration is needed to elucidate the influence of sample composition in complex matrices and the aggregation state of PS signal probes in the formation of nano‐lenses because these factors may affect the analytical capabilities of UL‐SNH. Variations in design parameters and types of SAW‐IDT chips may also influence the formation of the nano‐lens effect. Limited by the Abbe diffraction limit, achieving the simultaneous detection of multitargets with UL‐SNH remains challenging. To address these issues, the UL‐SNH biosensing platform will combine automated pre‐treatment devices, multiplexed detection capability and easily acquired ultra‐sensitive holographic imaging technology to achieve point‐of‐care testing in our future work.

## 3 Conclusion

We developed an unsupervised learning–assisted and SAW–IDT‐driven nano‐lens holography biosensing platform for the ultrasensitive and amplification‐free detection of viable bacteria. The nano‐lens–based holography microscope achieved the ultra‐sensitive holographic imaging of smaller‐sized PS microsphere signal probes, breaking through low SNR limitations and featuring an ultra‐wide FOV and high sensitivity. In addition, the unsupervised learning–assisted target detection algorithm can accurately detect PS microspheres and facilitate signal transduction after rapid training without manually labeled datasets and high‐configuration computers. Thus, our strategy integrates unsupervised learning and ultra‐sensitive holographic imaging technology into a biosensing platform with broad potential, which has numerous advantages over previous work and traditional qPCR methods in terms of time, cost, and accuracy. In the future, we will first continue to advance innovative nano‐lens–based holographic imaging strategies and biosensing technologies to enhance UL‐SNH for the simultaneous detection of multi‐pathogens. In addition, we will focus on developing portable automated pre‐processing devices and standardized sample preparation processes to overcome the current limitations of sample pre‐treatment. We believe our approach will contribute to the development of versatile holographic imaging biosensing technologies and even expand applications in cell ultra‐sensitive holographic imaging, disease in vitro diagnosis, and nanomaterial characterization.

## 4 Experimental Section

### SAW–IDT Chip Fabrication

The key parameter design and standard photolithographic fabrication of the SAW–IDT chip was based on published reports.^[^ ^23^, ^39^ ^]^ The customized IDT pattern was transferred to a 5‐inch quartz glass plate using UV lithography technology to create an SAW–IDT chip mask. Single‐crystal lithium niobate piezoelectric wafers (LiNbO_3_) were used as the substrate of SAW–IDT chips and the Rayleigh wave was propagated on piezoelectric 128° *Y*‐cut *X*‐propagating 4‐inch LiNbO_3_ with a thickness of 0.5 mm. SAW was generated by using a signal generator to generate a sine wave excitation signal of a specific frequency after amplification and inputting it into the IDT. The Rayleigh wave generated by IDT propagated on the LiNbO_3_ substrate and then coupled to the disposable glass coverslip through the gel to form the lamb wave. The design frequency of SAW–IDT was 9.71 MHz, determined by the dispersion curve of SAW propagating on a disposable cover glass, along with the initial phase velocity. The design wavelength was ∽411 µm, calculated by the frequency–wavelength formula, *λ* = *c*/*f*, where *f* is the design frequency, and *c* is the propagation speed of the SAW on the LiNbO_3_ substrate. The actual excitation frequency of the SAW‐IDT chip is influenced by manufacturing errors in the photolithography. The number of IDT titanium–gold electrode pairs was 40, and the aperture was 20 mm. The completed LiNbO_3_ substrate with electrodes was precisely cut into the specified shape and used for coupling to a disposable glass chip.

### Finite‐Element and Numerical Simulation

Finite‐element simulation of piezoelectric effects in SAW–IDT resonators and PS microsphere trajectories caused by SAW on the coverslip liquid cross section were carried out using COMSOL Multiphysics (version 6.0, COMSOL) using the 2D piezoelectric effect and acoustic flow domain coupling module, respectively. For the analysis of the SAW piezoelectric effect, the key parameters were set as follows: coordinate system–piezoelectric substrate 128° rotation coordinate system; static electricity–suspension potential, and periodic conditions of two sides; solid mechanic–fixed bottom, and periodic conditions of two sides; mesh size–extremely fine. For the analysis of PS microsphere trajectories, the key parameters were set as follows: thermoviscous acoustics, frequency domain–velocity of SAW; boundary condition–pressure of 0 Pa with suppression of backflow; particle tracing for fluid flow–acoustophoretic radiation force of SAW; mesh size–extremely fine. Numerical analysis of lamb wave dispersive curves for coverslip was carried out using the Vallen control panel analyzing software (version R2022.0808.3). The propagation of lamb wave in the liquid‐solid bilayer is based on the dispersion equation^[^ ^40^ ^]^

(1) $$\begin{array}{ccc} \pi^{2}\frac{\rho_{F}}{\rho }p\Omega^{4}[d^{2}\cos\left(2p\right)\sin\left(2q\right) & + & 4\xi^{2}pq\sin\left(2p\right)\cos\left(2q\right)]\sin\left(\frac{2ar}{h}\right)\;\\ & & +16rAS\cos(\frac{2ar}{h})=0 \end{array}$$

where

(2) $$A=d^{2}\sin\left(p\right)\cos\left(q\right)+4\xi^{2}pq\cos\left(p\right)\sin\left(q\right)$$

(3) $$S=d^{2}\cos\left(p\right)\sin\left(q\right)+4\xi^{2}pq\sin\left(p\right)\cos\left(q\right)$$

Equations (2) and (3) show the dispersion relations for the antisymmetric and symmetric guided modes (A and S mode) of the lamb wave propagation in the coverslip. Other parameter calculation methods were given by

(4) $$p=2\pi fh\sqrt{\frac{1}{c_{L}^{2}}-\frac{1}{v^{2}}}$$

(5) $$q=2\pi fh\sqrt{\frac{1}{c_{T}^{2}}-\frac{1}{v^{2}}}$$

(6) $$r=2\pi fh\sqrt{\frac{1}{c_{F}^{2}}-\frac{1}{v^{2}}}$$

(7) $$d=8\pi f^{2}h^{2}\left(\frac{1}{c_{T}^{2}}-\frac{2}{v^{2}}\right)$$

where Ω = 4*fh*/*c_T_*,ξ = 4*fh*/*v*, *f* represents the frequency, and *v* represents the lamb wave phase velocity. For the solid coverslip layer, 2*h*  represents the coverslip thickness (∽140 µm), ρ represents the glass mass density, $c_{L}$ and $c_{T}$ represent the velocities of longitudinal and transversal waves. For the liquid film layer, 2*a* represents the liquid film thickness, *ρ*_*F*_ represents the fluid mass density, $c_{F}$ represents the velocity of SAW in the liquid film.

### UL‐CNN Training

The UL–assisted image processing algorithm was developed based on the Deep‐Track framework (version 2.1)^[^ ^41^ ^]^ and built on TensorFlow (version 2.10.0),^[^ ^42^ ^]^ which was an object detection algorithm capable of rapid training with limited unlabeled datasets. This unsupervised object detection strategy proved that training on a single image was sufficient to satisfy the detection requirements under different complex conditions. The training dataset selected a single holographic image (512 × 512 pixels) and defined the characteristic image of a single holographic microsphere (80 × 80 pixels) as a training sample. These feature images used for training were subjected to affine transformation (rotation, translation, scaling, flipping), noise addition, Gaussian blur, and multiplication operations for data augmentation to create an adaptive training dataset. The training process was performed on a laptop equipped with an Intel i5–11400H CPU and an Nvidia GeForce GTX 1650 4GB GPU. After 30 epochs of training, the CNN was able to accurately identify the characteristic microspheres in other images and mark the center position of these target microspheres with “×” and output the counting number results of each image to calculate target concentrations simultaneously.

### Standard Protocol of UL‐SNH for *S. typhimurium* Detection

The detailed procedures of the UL‐SNH biosensing platform for detecting *Salmonella typhimurium* (*S. typhimurium*) were as follows: First, MNP–LPST10 phage conjugate solution (250 µg mL^−1^; 100 µL) was mixed with a sample solution containing *S. typhimurium* (1 × 10^0^−1 × 10^8^ CFU mL^−1^; 100 µL) for 15 min at room temperature to specifically capture viable target bacteria. After magnetic separation, the radioimmunoprecipitation assay (RIPA) lysis buffer (50 µL) was mixed with MNP–phage–bacteria complexes for 5 min to fully release the bacteria DNA. The other concentrations of *S. typhimurium* were added to MNP–phage conjugates according to this ratio. Second, Cas12a (2 µL; 1 µm), crRNA_1_ (2 µL; 1 µm), crRNA_2_ (2 µL; 1 µm), and were mixed with NE buffer 2.1 (1 ×; 4 µL) in a centrifuge tube for 15 min at 37 °C to assemble RNPs. This procedure occurred simultaneously with phage–mediated viable bacteria DNA extraction. Then, the assembled RNPs (9 µL), MNP–ssDNA conjugate solution (80 µg mL^−1^; 9 µL), *S. typhimurium* DNA (9 µL), and NE buffer 2.1 (10 ×; 3 µL) were added to a centrifuge tube for 60 min at 37 °C to trans‐cleavage ssDNA probes. After the CRISPR reaction, the trans‐cleaved MNP–ssDNA substrate was magnetically separated and washed thrice with deionized water. Then, PS–SA conjugates (200 µg mL^−1^; 20 µL) were added to the trans‐cleaved MNP–ssDNA substrate for 10 min at 37 °C. After magnetic separation, the supernatant (15 µL) was mixed with polyethylene glycol–600 (PEG–600; 15 µL) to retain for holographic imaging. Third, each sample droplet (∽8 µL) was spread evenly onto a pre‐treated disposable quartz coverslip, which was washed and dried to ensure they were not contaminated by impurities. In addition, these disposable quartz coverslips were plasma‐treated for 5 min before use to render them hydrophilic, resulting in a quicker formation of a thin layer of a sample droplet covering the surface. The coverslip and SAW–IDT chip were coupled through the gel, and the liquid layer of the coverslip was inverted onto the CMOS sensing area. Finally, the CMOS image sensor captured holograms (3840 × 2160 pixels) according to the Poisson sampling method, and three droplets of each sample were measured three times.

### Statistical Analysis

Statistical analysis was performed using GraphPad Prism version 9.5.1 for Windows (GraphPad Software, San Diego, California USA, www.graphpad.com). All data were represented as the mean ± standard deviation (SD), unless noted otherwise. No statistical methods were used to pre‐determine the sample size, which was described in the context or figure captions. For two‐group comparisons, statistical significance was assessed using the unpaired two‐tailed Student's *t*‐test with *α *= 0.05. Statistical significance was set at *P* < 0.05.

## Conflict of Interest

The authors declare no conflict of interest.

## Supporting information



Supporting Information

## Data Availability

The data that support the findings of this study are available from the corresponding author upon reasonable request.
