# Supplementary material for: Unsupervised Learning‐Assisted Acoustic‐Driven Nano‐Lens Holography for the Ultrasensitive and Amplification‐Free Detection of Viable Bacteria
Source: Adv Sci (Weinh). 2024 Nov 22;12(2):2406912. doi: 10.1002/advs.202406912 (PMC11727406; doi:10.1002/advs.202406912)
Supplement: Supplementary file 1 — Supporting Information [file ADVS-12-2406912-s001.docx]

**Supporting Information for**

**Unsupervised Learning-Assisted Acoustic-Driven Nano-Lens Holography for the Ultrasensitive and Amplification-Free Detection of Viable Bacteria**

Authors:

Yang Zhou, Junpeng Zhao, Junping Wen, Ziyan Wu, Yongzhen Dong, Yiping Chen*

**Content**

[Materials 4](#_Toc181711406)

[Apparatus 4](#_Toc181711407)

[Preparation of PS–SA, MNP_1000_–phage, and MNP_1000_–ssDNA–biotin 5](#_Toc181711408)

[Bacteria Culture 6](#_Toc181711409)

[qPCR Detection 6](#_Toc181711410)

[Real Sample Analysis 7](#_Toc181711411)

[Figure S1 9](#_Toc181711412)

[Figure S2 10](#_Toc181711413)

[Figure S3 11](#_Toc181711414)

[Figure S4 12](#_Toc181711415)

[Figure S5 13](#_Toc181711416)

[Figure S6 14](#_Toc181711417)

[Figure S7 15](#_Toc181711418)

[Figure S8 16](#_Toc181711419)

[Figure S9 17](#_Toc181711420)

[Figure S10 18](#_Toc181711421)

[Figure S11 19](#_Toc181711422)

[Figure S12 20](#_Toc181711423)

[Figure S13 21](#_Toc181711424)

[Table S1 22](#_Toc181711425)

[Table S2 23](#_Toc181711426)

[Table S3 24](#_Toc181711427)

[Table S4 25](#_Toc181711428)

[Table S5 26](#_Toc181711429)

[Table S6 27](#_Toc181711430)

[Table S7 28](#_Toc181711431)

[References 29](#_Toc181711432)

Materials**.** The carboxyl group-functionalized PS microspheres (PS–COOH,99 nm, 100 mg/mL, PC02N; 510 nm, 100 mg/mL, PC03001; 907 nm, 100 mg/mL, PC03004; 3 μm, 100 mg/mL, PC05003; 5.98 μm, 100 mg/mL, PC06003) were purchased from Bangs Laboratories, Inc (USA). MNP–NH_2_ (1 μm, 10 mg/mL, XFJ109) was purchased from XFNANO Biological Technology Co., Ltd (Jiangsu, China). Recombinant streptavidin (Cat No. P5084, 18.29 U/mg, 1 mg) was purchased from Beyotime Biotechnology (Shanghai, China). 1–Ethyl–3– (3–dimethyl aminopropyl)–carbodiimide hydrochloride (EDC), Hydroxy–2, 5–dioxopyrrolidine–3–sulfonic acid sodium salt (Sulfo–NHS), 2–morpholinoethanesulphonic acid (MES), and polyethylene glycol (PEG–600) were purchased from Aladdin (Shanghai, China). Bovine serum albumin (BSA) and tween–20 were purchased from Amresco (USA). The buffers used in the experiment include phosphate-buffered saline (PBS, 0.01 M, pH 7.4), PBST (0.01 M, PBS containing 0.05% Tween–20), MES (0.1 M, pH 6.0), and MEST (0.01 M, MES containing 0.05% Tween–20). Engen® Lba Cas12a (Cpf1, 100 µM) and NEB buffer 2.1 (10 ×, Lot: 10149688) were purchased from New England Biolabs (USA). The 20 bp DNA Ladder was purchased from Takara Biomedical Technology Co., Ltd (Beijing, China). The Hieff Unicon® qPCR TaqMan probe master mix was purchased from Yeasen Biotech Co., Ltd (Shanghai, China). The diethylpyrocarbonate (DEPC) water and all DNA/RNA sequences (**Table S1**) were synthesized by Sangon Biotech Co., Ltd. (Shanghai, China). *Escherichia coli* (*E. coli*, ATCC 43888), *Listeria monocytogenes* (*L. monocytogenes*, ATCC 19114), *Staphylococcus aureus* (*S. aureus*, ATCC 29213), and *Salmonella* *Typhimurium* (*S*. *typhimurium*, ATCC 14028) were purchased from American Type Culture Collection (Manassas, VA, USA). All food samples were purchased from local supermarkets at Huazhong Agricultural University (Wuhan China). The water used for the experiments was deionized by a water purification system (Millipore, USA). All of the chemicals were of analytical grade and used without further purification.

Apparatus**.** The CMOS sensor was purchased from IDS Imaging Development Systems GmbH (UI-3592LE-C Rev.2, AB02301, Obersulm, Germany) and the partially coherent light source was purchased from HAYEAR (Shenzhen, China). The function signal generator was purchased from RIGOL Technologies Co., Ltd (DG5352, 350MHz, Suzhou, China), and the amplifier was purchased from Feiyi Technologies Co., Ltd (FPA301, Zhengzhou, China). The DC power supply was purchased from MaiSheng Technologies Co., Ltd (MP1005D, Shenzhen China). The vacuum plasma surface treatment system was purchased from PTL Electrical Technologies Co., Ltd (PTL-VR500, Zhaoyuan, China). The field emission scanning electron microscope (TESCAN MIRA4, Czech) was used to characterize the immunocomplex. The Super-Mag separator was purchased from Ocean Nano-Tech (USA). A multifunctional enzyme marker (Bio-Tek, Synergy H4) was employed to detect fluorescence signals. The fluorescence monitoring assay was performed on the real-time PCR System (Jena, Qtower 2.2). An optical microscope (DM-3000, LEICA, Germany) was employed to compare with the CMOS sensor.

Preparation of PS–SA, MNP_1000_–phage, and MNP_1000_–ssDNA–biotin **conjugates.** The coupling strategy for PS microspheres and MNP with different biometric molecules was according to our published work and the standard manual provided by reagent manufacturers^[1]^. The preparation of PS–streptavidin conjugates (PS–SA) was as follows: Firstly, PS solution (1 mg) was added to a 1.5 mL tube and washed with MEST three times. After that, EDC (10 μL, 5 mg/mL) and NHS (10 μL, 5 mg/mL) were added to mix with the PS solution to activate the carboxyl group. The mixture was diluted to 1 mL with MES and mixed at room temperature for 15 min at slow rotation. The activated PS solution was washed three times with PBST (1 mL) and centrifuged for 5 min at a speed of 6, 000 r/min. Secondly, SA (30 μg) was added to the tube and diluted to 500 μL by PBS. The mixture was coupled for 30 min at 37 ℃ with rotation slowly to make an adequate reaction. After that, PBST (1 mL, containing 1% BSA, pH 7.4) was added to mix with PS–SA conjugates to block the residual active sites for 30 min. Thirdly, the blocked PS–SA conjugates were washed with PBST (1 mL) three times and resuspended by PBST (1 mL, containing 0.5% BSA, 0.02% NaN_3_) to store at 4 ℃. The preparation of MNP–phage conjugates was similar to the above, the difference was that centrifugation was replaced by magnetic separation. The usage of EDC (20 μL, 10 mg/mL) and NHS (10 μL, 10 mg/mL) was different for coupling MNP–phage conjugates (LPST10, 40 μL, 10^9^ PFU/mL). The coupling process was at room temperature for 3.5 h to store at 4 ℃.

The preparation of MNP_1000_–ssDNA–biotin conjugates was as follows: Firstly, EDC (5 μL, 5 mg/mL) and NHS (5 μL, 5 mg/mL) were added to mix with the biotin–ssDNA–COOH solution (10 μL, 50 μM) at 37℃ for 15 min to activate the COOH group. Secondly, the above-activated ssDNA solution was added to the amine-modified MNP solution (1 mL, 1 mg/mL) with gently shaken at room temperature for 3 h to form MNP–ssDNA–biotin conjugates. Finally, the unreacted biotin–ssDNA–COOH solution was removed by magnetic separation, which was repeated 3 times. The MNP_1000_–ssDNA–biotin conjugates were resuspended in 500 μL of PBS buffer to store at 4 ℃.

Bacteria Culture**.** The bacterial strains used in this study obtained from the American type cells collection (ATCC): *Escherichia coli* (*E. coli*) (ATCC 43888), *Listeria monocytogenes* (*L. monocytogenes*, ATCC 19114), *Staphylococcus aureus* (*S. aureus*, ATCC 29213), and *Salmonella* Typhimurium (*S. typhimurium*, ATCC 14028). Four types of bacteria were added to the TSB medium (50 mL) and incubated at 37 °C overnight in an orbital shaker incubator. The microbial solution was then transferred to a new TSB medium and incubated until the OD_650_ value was within the range of 0.6 to 1. The concentration of bacteria was determined using the plate counting method, where serial dilution solutions (0.1 mL) were spread onto corresponding agar plates. The agar plates were inverted and incubated at 37 °C for 24 h, and the colonies on plates bearing 30 to 300 colonies were subsequently counted. The concentration of the bacterial solution was calculated based on the dilution factor.

qPCR Detection**.** The qPCR detection steps were carried out according to the following Chinese industry standards and analytical testing society standards (SN/T 1870–2016). The probe method was selected for qPCR detection of *S. typhimurium*. The Hieff Unicon® qPCR TaqMan probe master mix (10 μL, 1 ×), PCR forward primer (0.4 μL, 10 μM), PCR reverse primer (0.4 μL, 10 μM), qPCR FAM probe (0.2 μL, 10 μM), target DNA (2 μL), and sterile ultrapure water (7 μL) were mixed into a centrifuge tube for each sample. The PCR reaction was initiated by pre-denaturation at 95°C for 3 min, followed by denaturation at 95°C for 5 s. The annealing and extension step was performed at 60°C for 40 s while collecting FAM fluorescence for 40 cycles and the reaction product was then stored at 4°C.

Real Sample Analysis**.** For artificially contaminated food analysis, the samples were purchased from local supermarkets near Huazhong Agricultural University (Wuhan, China) and processed in accordance with the Chinese national standards (GB 4789.4-2016). After pre-treatment and static settlement for 10 min, the supernatants of these samples were carefully preserved and subsequently cultured in a selective medium to ensure that it was free of *S. typhimurium.* There were a total of 15 samples in each category, including 12 suspected contaminated food samples and 3 non-bacterial samples (high-temperature sterilized). In the suspected contamination sample group, each concentration of *S. typhimurium* ranging from 10^2^ to 10^7^ CFU/mL was employed to prepare artificially contaminated food samples. For each category, these *S. typhimurium*-contaminated positive samples were combined with several negative samples (not sterilized) to be made into 15 blind food samples for UL-SNH and qPCR detection.

For clinical sample analysis, samples were provided by the General Hospital of Central Theater Command (Wuhan, China, 2023-026-01) from patients suspected of foodborne pathogen infection during microbiological examinations. These samples included urine, serum, stool, and other types. A sterile loop was used to collect these samples, which were then deposited on the XDL selective medium and incubated overnight at 37°C. After standard culturing, all samples were identified to confirm if they were *S. typhimurium* positive or negative, assuring that the samples were not contaminated by other concurrent bacteria. Subsequently, colonies on the culture medium were rinsed with sterile water, and 1 mL of the rinse solution was collected and centrifuged at 12,000 rpm for 5 min. The supernatant was discarded, and the resulting pellet was resuspended, repeating the process twice. A total of 15 clinical sample pre-treatment processes were conducted in the hospital and then provided for direct analysis. These samples were detected by both UL-SNH and qPCR methods to further evaluate the analysis capabilities of our approach. It should be noted that the UL-SNH method used a phage-mediated DNA extraction method, while qPCR employed a commercial DNA extraction kit to extract nucleic acids. The genomic DNA was extracted using a bacteria genomic DNA extraction kit according to the manufacturer’s instructions and stored at −20 °C until being used.

The pre-treatment steps of solid food samples were taken as follows: briefly, chopped solid real samples (25 g) were transferred into a sterile plastic bag containing 225 mL of sterile BPW buffer, and homogenized for 10 min at 10000 rpm/min. Subsequently, the supernatant of samples (100 μL) was taken for both UL-SNH biosensing and qPCR method detection after standing for 10 min. For the detection of *S. typhimurium* in liquid samples like milk, the sample (100 μL) was diluted 10-fold using sterile water before being subjected to various detection methods. For other food samples, the supernatant can be taken for detection after pre-treatment as described above. The average PS microsphere number in the supernatant and Ct value corresponding to each sample were substituted into the standard curve model to calculate the *S. typhimurium* concentration. The concentration of *S. typhimurium* in the samples (CFU/g) = (Calculated *S. typhimurium* from the equation CFU/mL × 225 mL)/25 g = 9 × Calculated *S. typhimurium* from the equation (CFU/g), according to the pre-treatment process of solid food samples. The concentration of *S. typhimurium* in the samples (CFU/mL) = 10 × [calculated *S. typhimurium* from the equation (CFU/mL)], according to the pre-treatment process of liquid food samples.


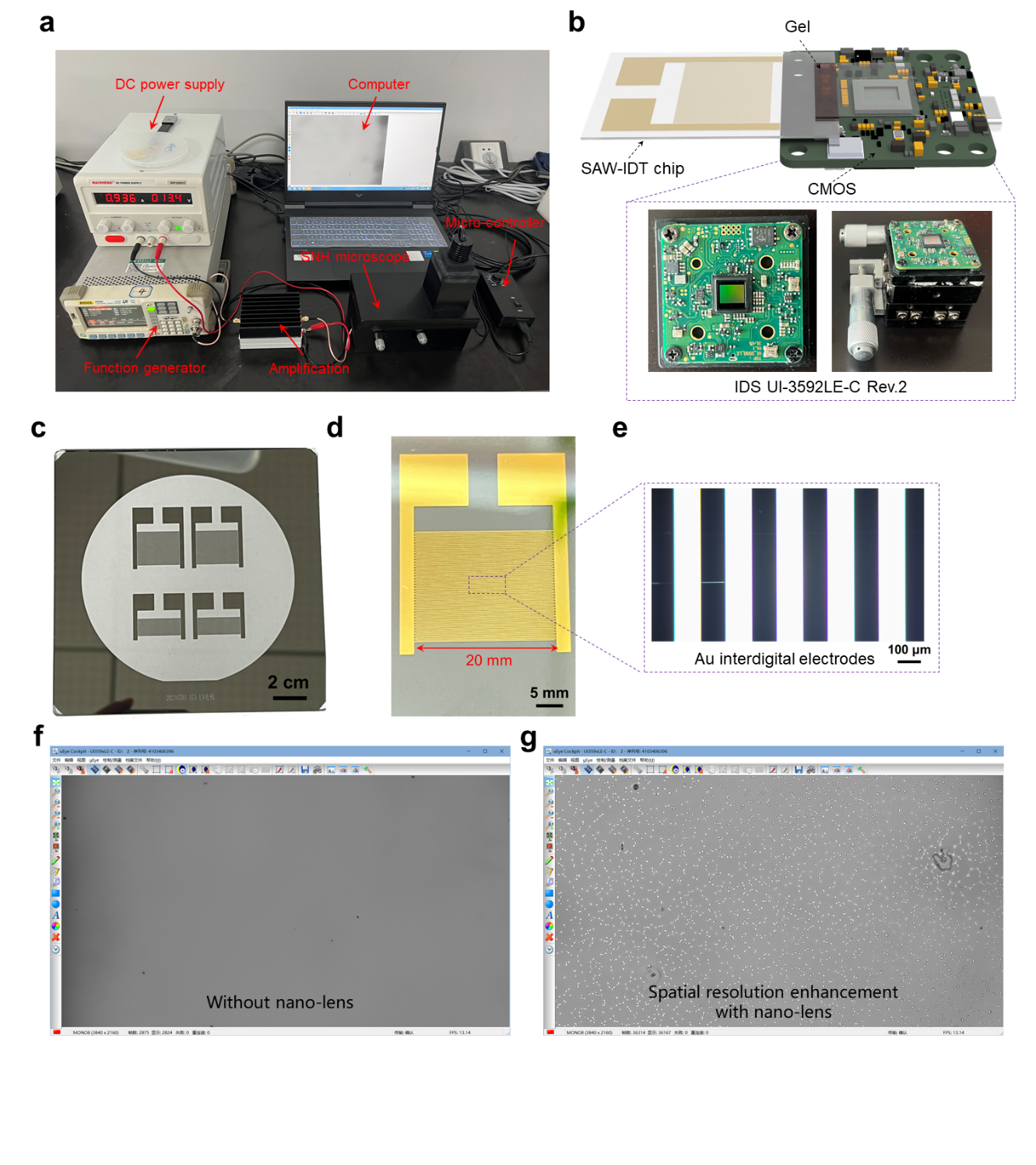


Figure S1. Construction and SAW-IDT resonator characterization of UL-SNH biosensing platform. a) Construction of UL-SNH biosensing platform, including an SNH microscope, DC power supply, function generator, amplification, micro-controller, and computer. b) Structure between CMOS sensor and SAW-IDT resonator. c) Photolithography mask plate of SAW-IDT resonator. d) Structure of SAW-IDT resonator with an acoustic aperture value of 20 mm. e) Characterization of SAW-IDT resonator electrodes by an optical microscope. IDS image acquisition software of 3 μm PS microspheres f) without and g) with nano-lens spatial resolution enhancement.


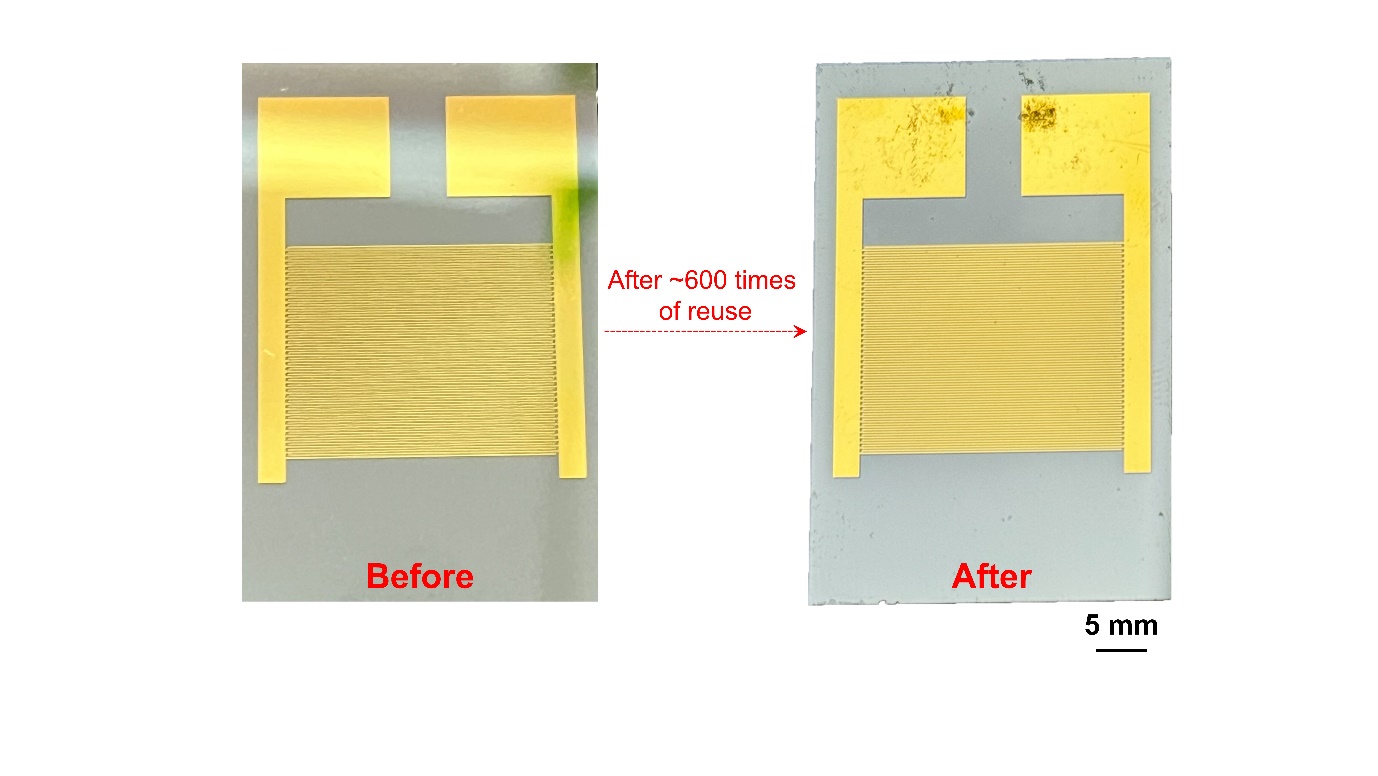


Figure S2. Comparison of SAW-IDT chip before and after reuse ~600 times.


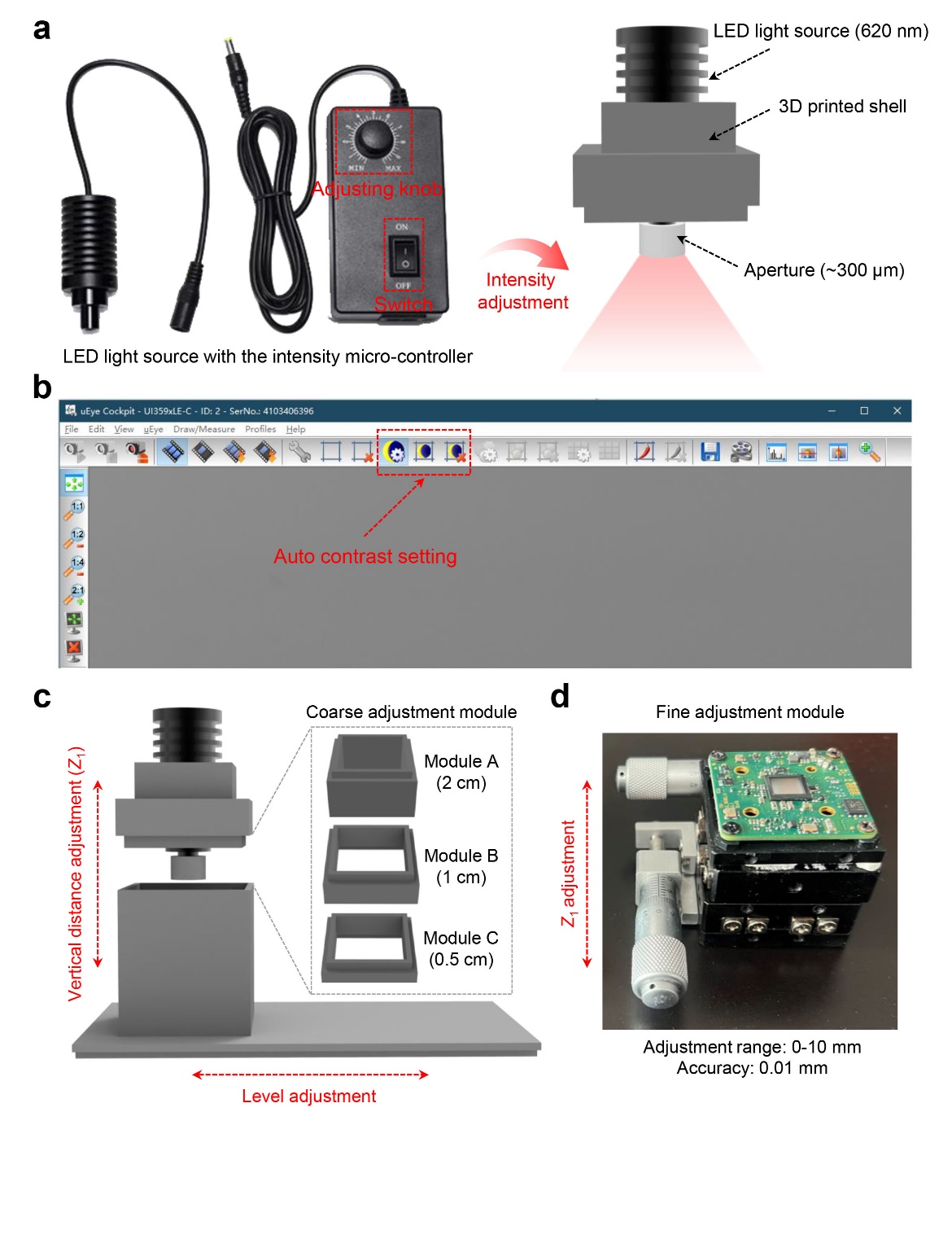


Figure S3**.** Parameter optimization of SNH microscope, including light intensity and working distances. a) LED light source with a micro-controller for intensity adjustments. b) IDS CMOS sensor with supporting driver software (IDS uEye Cockpit) for automatic contrast settings. c) SNH microscope with coarse adjustment modules (2, 1, and 0.5 cm) for vertical distance adjustments. d) IDS CMOS sensor with fine adjustment module for *Z*_1_​ height adjustment (0-10 mm, accuracy: 0.01 mm).


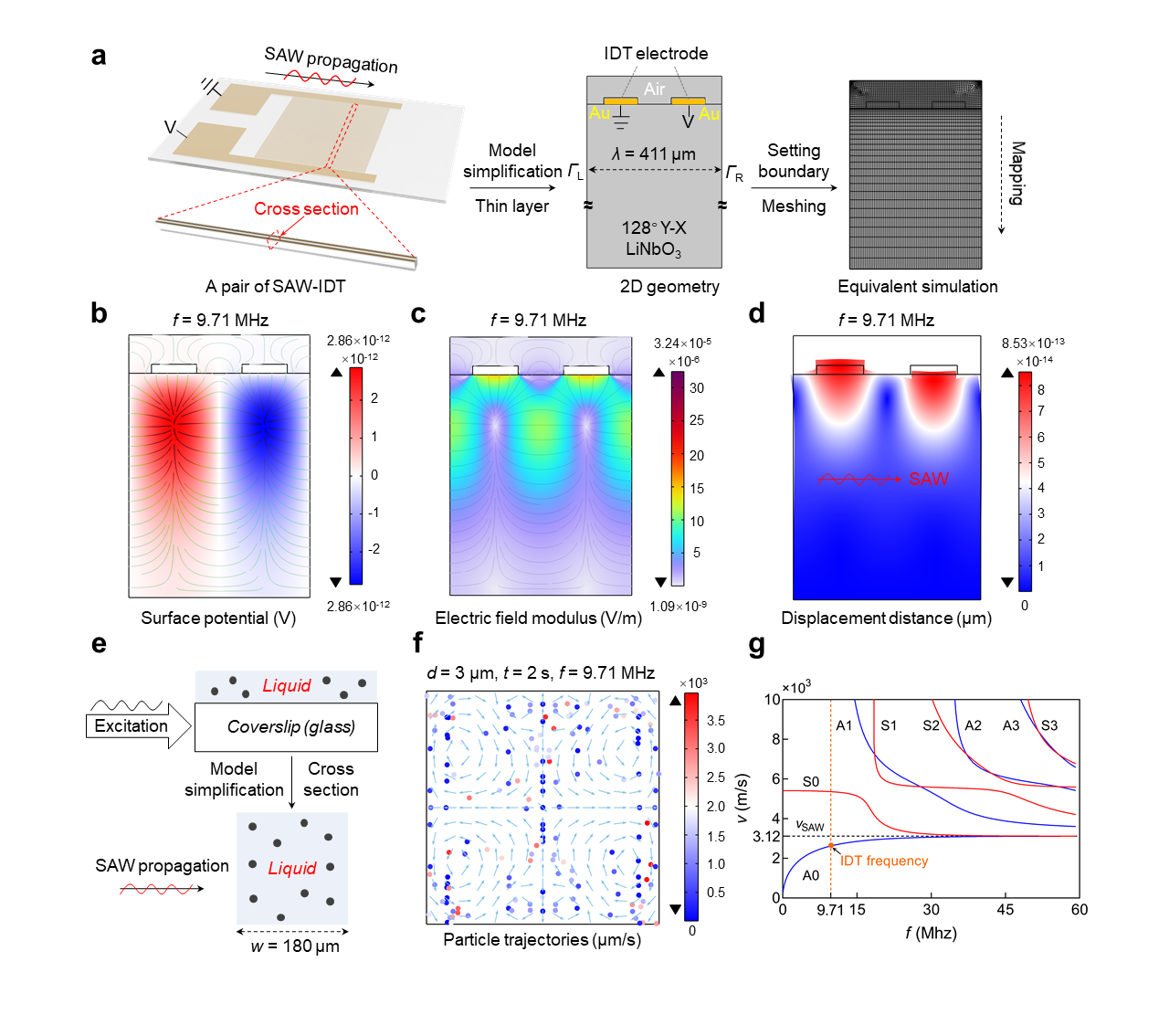


Figure S4. Multiphysics coupling simulation and numerical analysis. a) Schematic of the simplified SAW–IDT resonator 2D geometry. b) Surface potential distribution, c) electric field modulus, and d) vibration displacement distance of SAW–IDT resonator at 9.71MHz characteristic frequency. e) Schematic of simplified fluid–solid coupling simulation 2D geometry. f) Simulation of 3 μm PS microsphere trajectories in the liquid layer. g) Dispersion curves for coverslip.


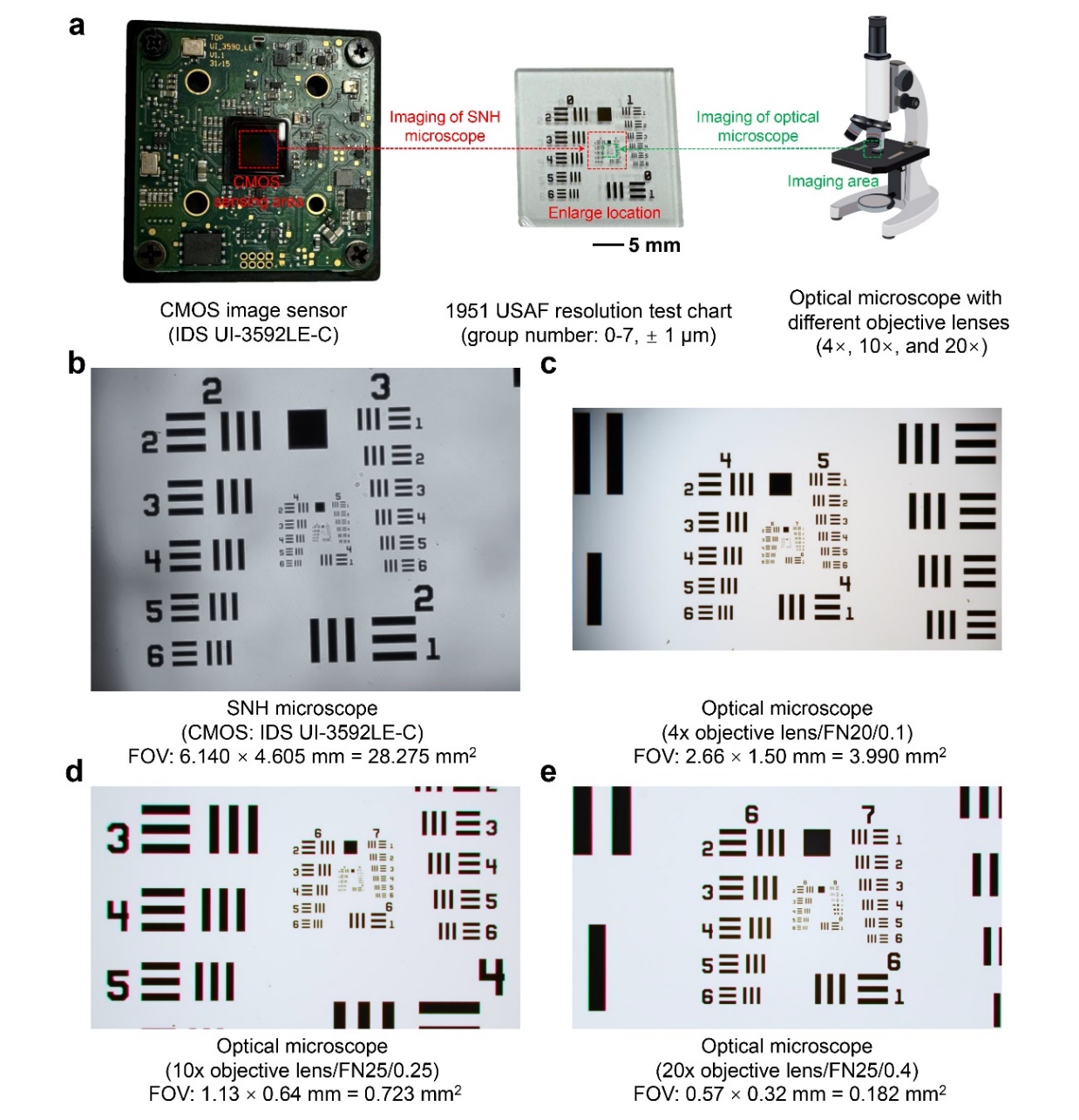


Figure S5**.** Comparison of FOV between SNH microscope and conventional optical microscope with different objective lenses (4×, 10×, and 20×) tested by the 1951 USAF resolution test chart (group number: 0–7, ± 1 μm). a) Schematic of the SNH microscope and optical microscope tested using the 1951 USAF resolution test chart. Testing result of b) SNH microscope without objective lens (visible group number: 2–3, 4912 × 3684 pixels). Testing results of a conventional optical microscope with c) 4× (visible group number: 4–5, 3840 × 2160 pixels), d) 10× (visible group number: 6–7, 3840 × 2160 pixels), and e) 20 × objective lenses (visible group number: 6–7, 3840 × 2160 pixels), respectively.


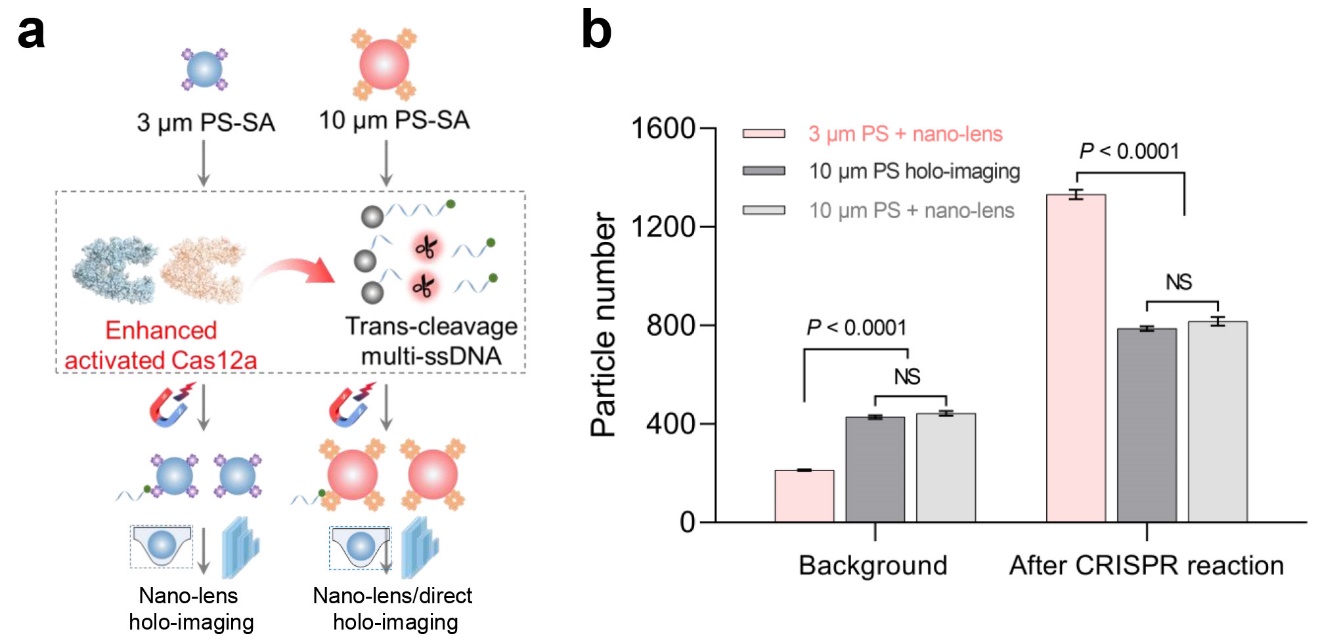


Figure S6. Comparison of PS microsphere probes with different particle sizes (3 μm and 10 μm) in the CRISPR reaction. a) Schematic of PS microsphere probes of different particle sizes employed in the CRISPR reaction. b) Comparative analysis of PS microsphere signal readout results between 3 μm and 10 μm using UL-SNH at *α* = 0.05. *n* = 3 technical replicates, unpaired two‐tailed Student's *t*‐test, error bars represent mean ± SD.


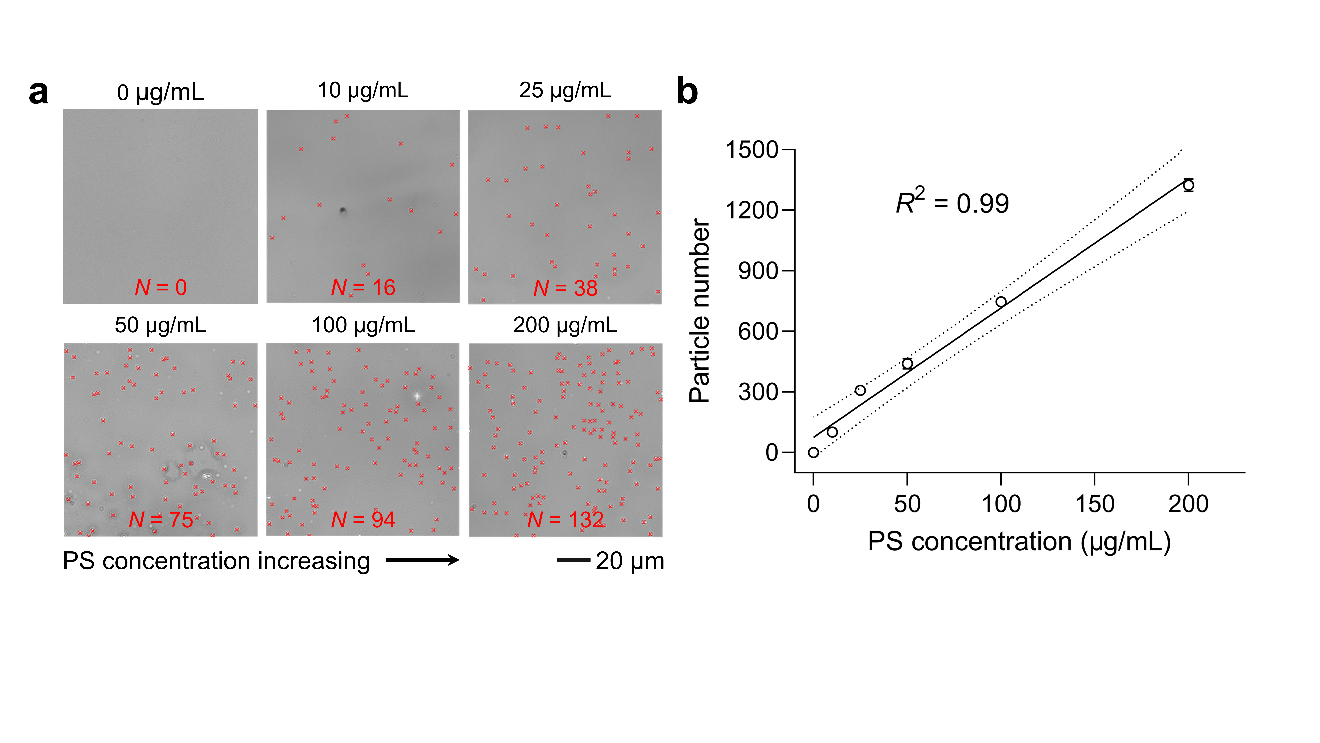


Figure S7. Evaluation of SNH microscope for 3 μm PS microsphere nano-lens imaging. a) UL-CNN-identified images of pure PS microspheres with different concentrations captured by the SNH microscope (1024 × 1024 pixels). b) Correlation analysis of the particle number with different pure PS microsphere concentrations (95% confidence interval). *n* = 3 technical replicates, error bars represent mean ± SD.


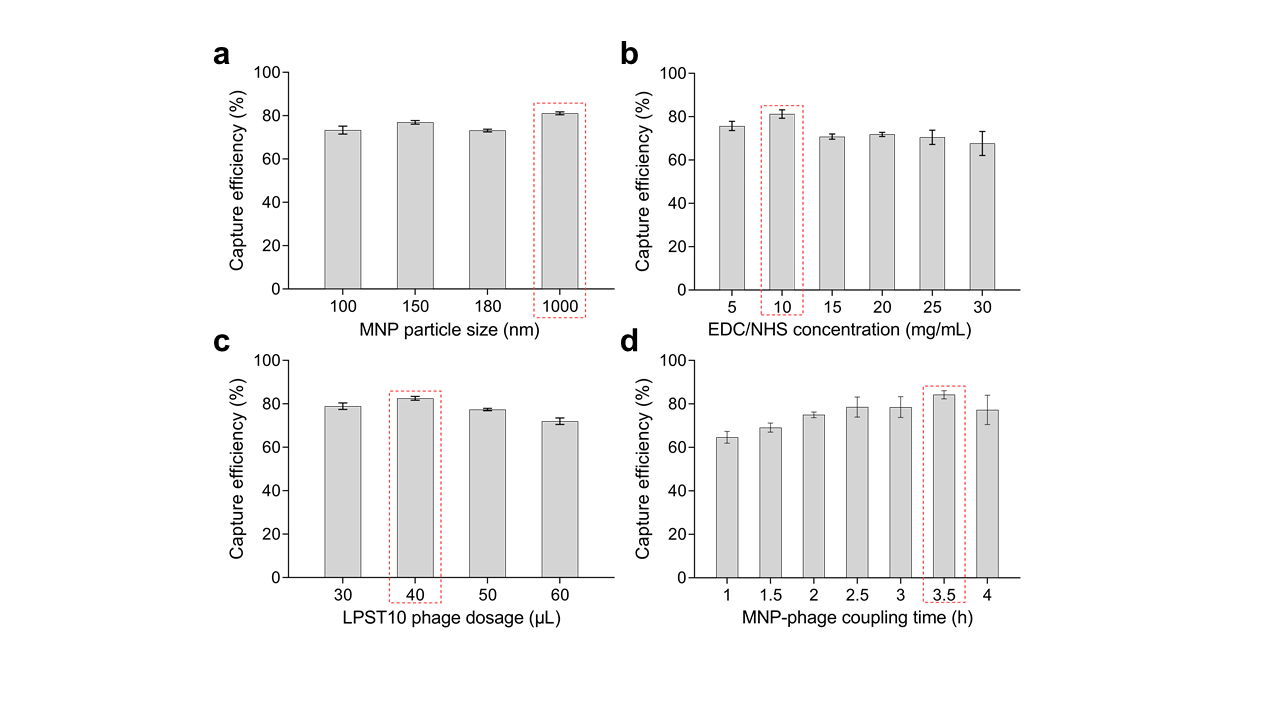


Figure S8. Parameter optimization of conjugation between MNP and LPST10 phage, including a) MNP particle size (100, 150, 180, and 100 nm), b) EDC/NHS concentration (5, 10, 15, 20, 25, and 30 mg/mL), c) LPST10 phage dosage (30, 40, 50, and 60 μL), and d) MNP–phage coupling time (1, 1.5, 2, 2.5, 3, 3.5, and 4 h). *n* = 3 technical replicates, error bars represent mean ± SD.


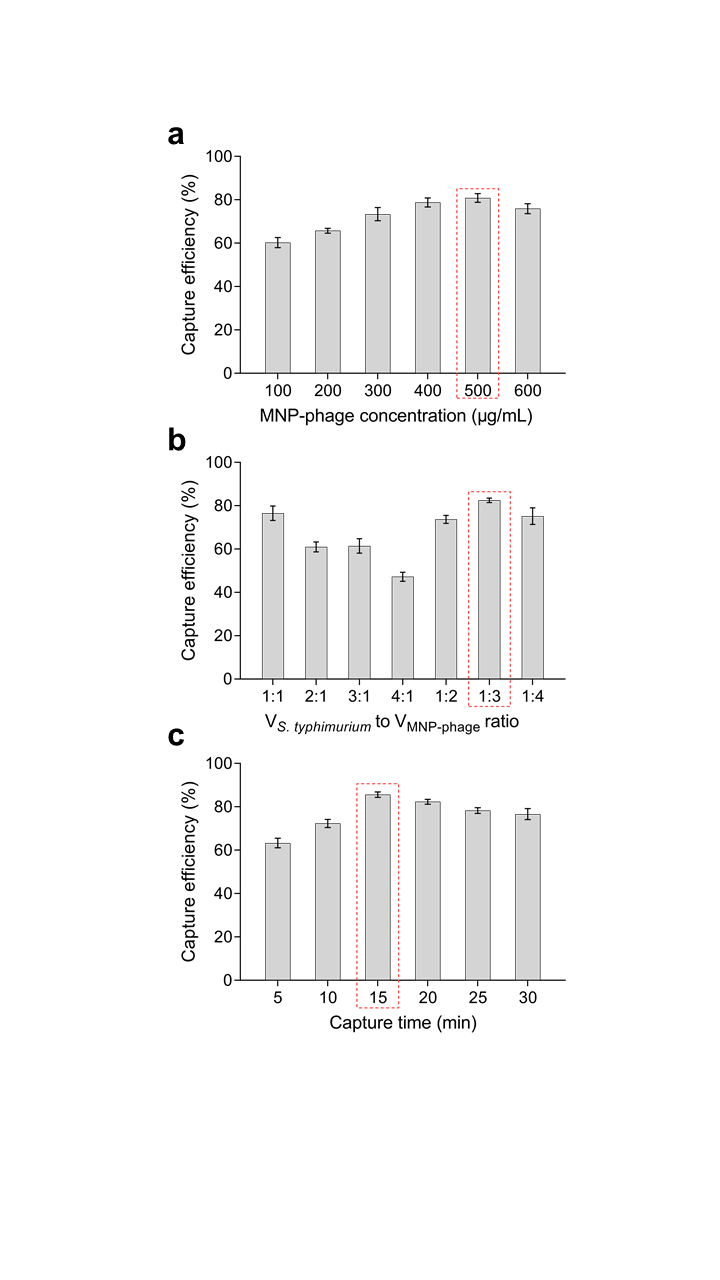


Figure S9. Parameter optimization of phage-mediated DNA extraction, including a) MNP–phage concentration (100, 200, 300, 400, 500, and 600 μg/mL), b) volume ratio of *S. typhimurium* to MNP–phage (1: 1, 2: 1, 3: 1, 4: 1, 1: 2, 1: 3, and 1: 4), and c) capture time (5, 10, 15, 20, 25, and 30 min). *n* = 3 technical replicates, error bars represent mean ± SD.


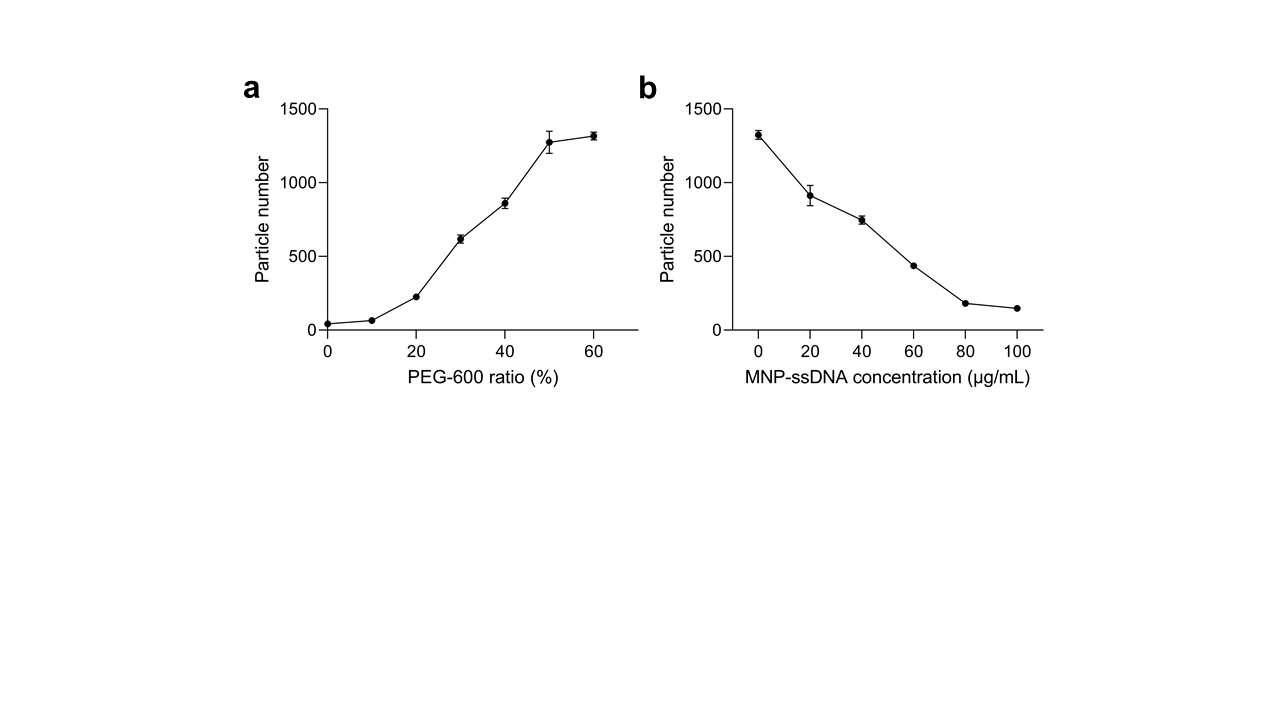


Figure S10. Parameter optimization of 3 μm PS microsphere nano-lens formation and background calibration, including a) PEG–600 ratio (0, 10, 20, 30, 40, 50, and 60%) and b) MNP–ssDNA concentration (0, 20, 40, 60, 80, and 100 μg/mL). *n* = 3 technical replicates, error bars represent mean ± SD.


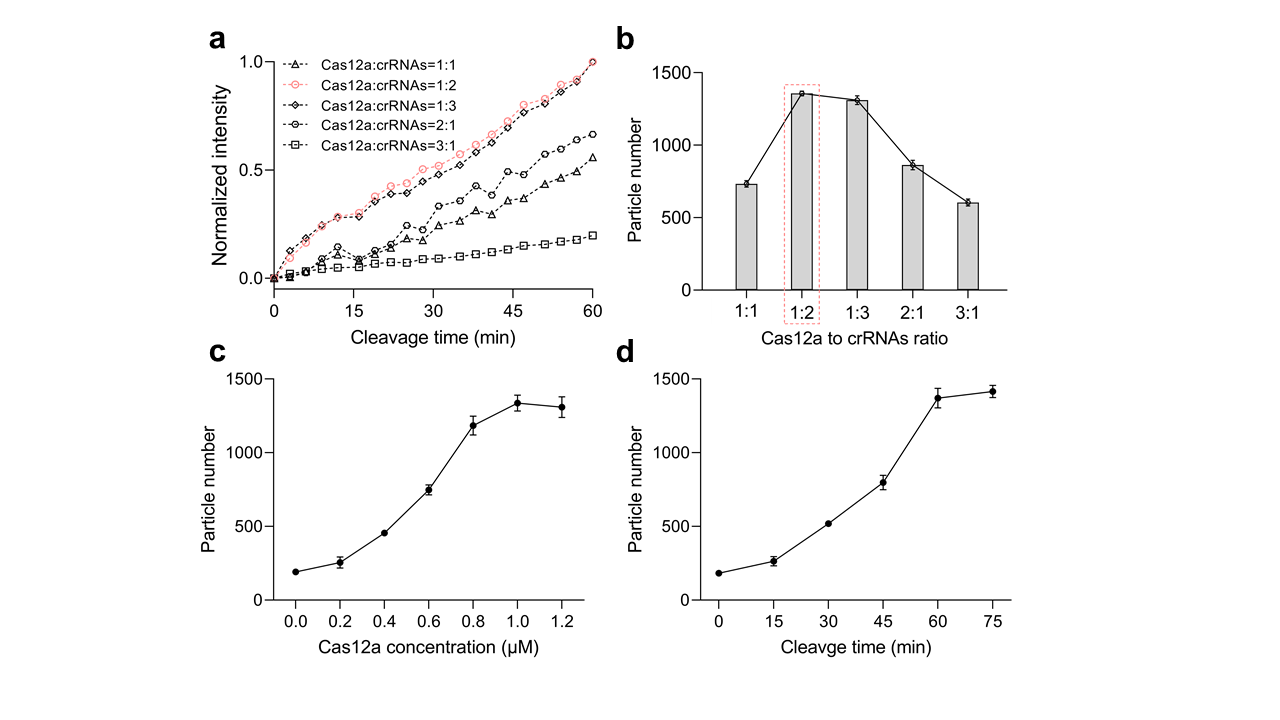


Figure S11. Parameter optimization of enhanced CRISPR-Cas12a system, including a, b) the ratio of Cas12a to crRNAs (1: 1, 1: 2, 1: 3, 2: 1, and 3: 1), c) Cas12a concentration (0, 0.2, 0.4, 0.8, 0.8, 1.0, and 1.2 μM), and d) cleavage time (0, 15, 30, 45, 60, and 75 min). *n* = 3 technical replicates, error bars represent mean ± SD.


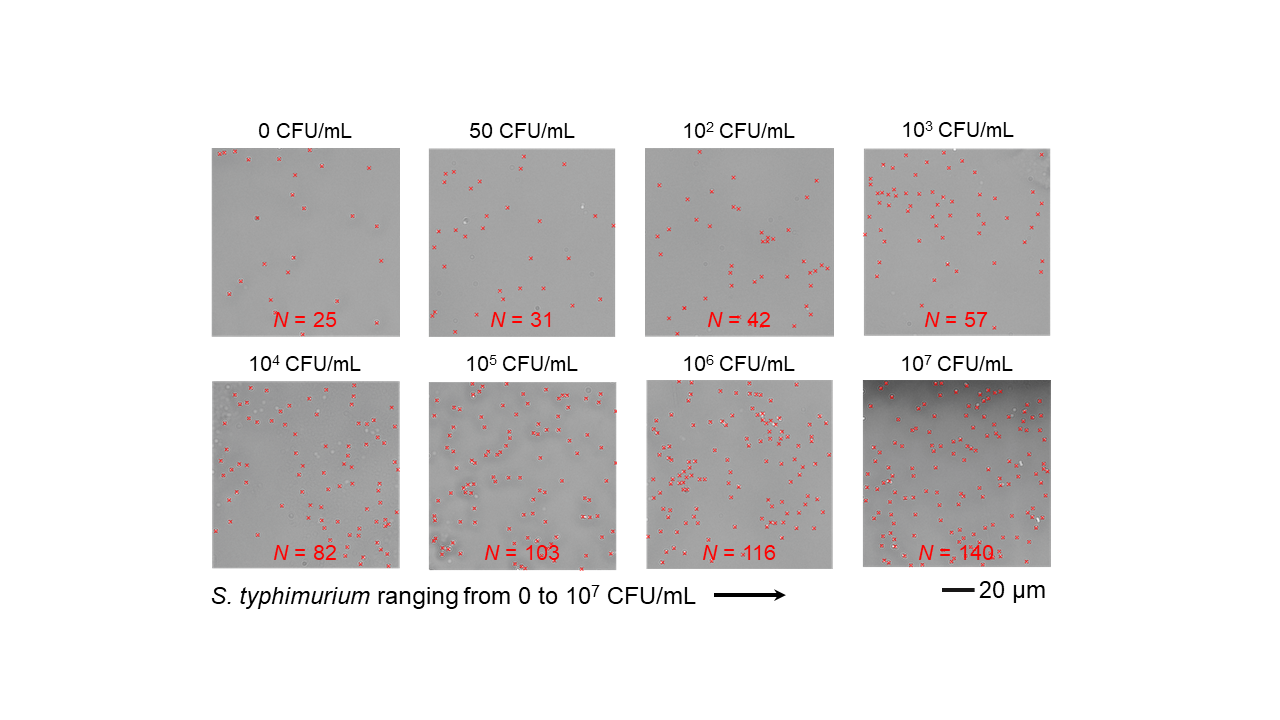


Figure S12. Holograms of PS microsphere in the supernatant for detection of *S. typhimurium* by UL-SNH biosensing platform (1024 × 1024 pixels).


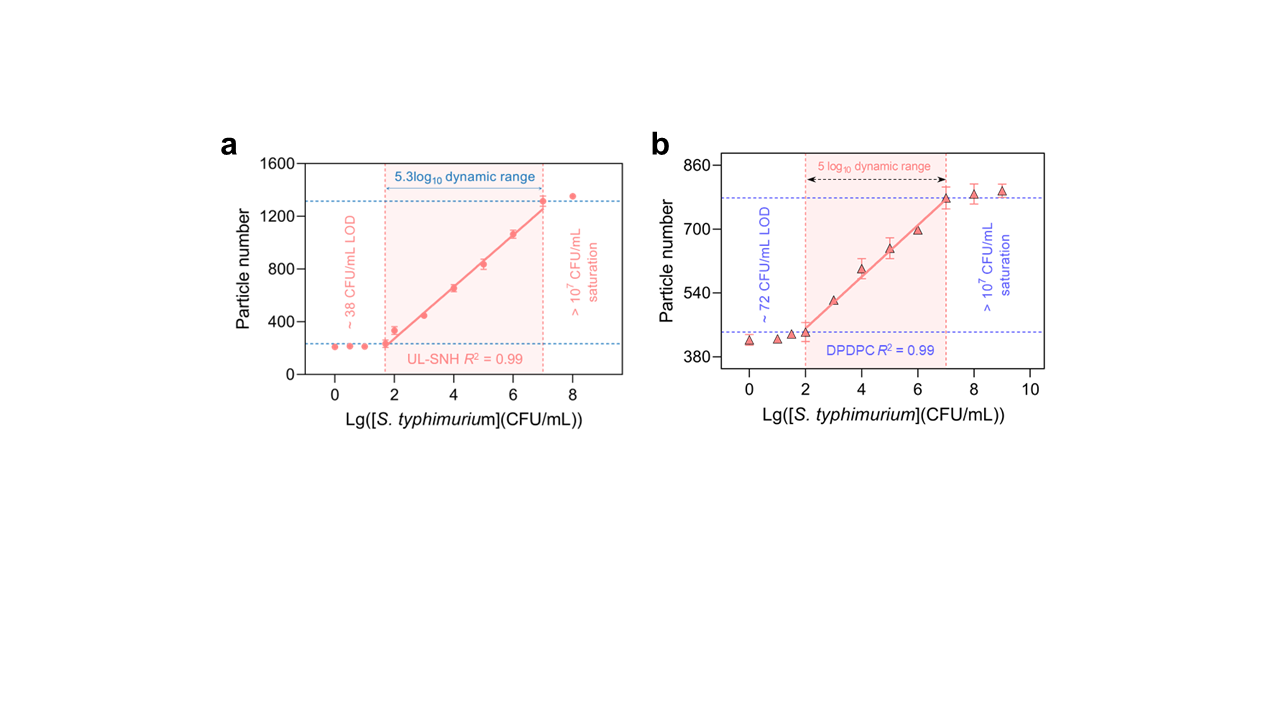


Figure S13. Performance comparison of UL-SNH (this work) and DPDPC (our previous work^[2]^ ) biosensing platform for *S. typhimurium* detection. *n* = 3 technical replicates, error bars represent mean ± SD.

Table S1. DNA and RNA sequences used in this work.

| Name | Sequence (5´– 3´) | Length (nt) |
| --- | --- | --- |
| COOH–ssDNA–biotin | Biotin–TTAAATTTAATTTAAATTTAATATT–COOH | 25 |
| crRNA_1_ | UAAUUUCUACUAAGUGUAGAUCCGGGCAUACCAUCCAGAGAAAA | 44 |
| crRNA_2_ | UAAUUUCUACUAAGUGUAGAUGUCUGGCAUUAUCGAUCAGUACC | 44 |
| FAM probe | (6–FAM)–ACTATAGGCTGGTT–BHQ_1_ | 14 |
| PCR forward primer | GCGGCGTTGGAGAGTGATA | 19 |
| PCR reverse primer | AGCAATGGAAAAAGCAGGATG | 21 |
| qPCR FAM probe | (6–FAM)–CATTTCTTAAACGGCGGTGTCTTTCCCT–BHQ_1_ | 28 |

Table S2. Comparison of SNH microscope and portable lens-free holographic microscope.

| **Category** | **SNH microscope**  **(This work)** | **Portable lens-free holographic microscope (Previous work)** |
| --- | --- | --- |
| Imaging strategy | Nano-lens imaging | Directly imaging |
| FOV (mm^2^) | ~ 28.28 | ~ 24.39 |
| Sensitivity | ≥ 99 nm | ≥ 10 μm |
| Distance adjustment | Coarse/fine adjustments | – |
| SNR enhancement | Nano-lens enhancement | – |
| Light intensity adjustment | CMOS adaptive contrast | – |
| Image size | 4912×3684 (4K) | 2560×1920 (2K) |

Table S3. Comparison of UL-CNN and YOLOv7-based image processing algorithm.

| **Method** | **UL-CNN**  **(This work)** | **ASP+YOLOv7**  **(Previous work)** |
| --- | --- | --- |
| Category | Unsupervised learning | Supervised deep learning |
| Detection principle | Geometric transformation | A single end-to-end network |
| Training dataset | A single image without manual labels | 1100 pairs of pair images with manual labels |
| Training device | CUP: Intel i5–11400 H  GPU: Nvidia GTX 1650 | CUP: Intel i9–12900 K  GPU: Nvidia RTX 3090ti |
| GPU bandwidth | 128 GB/s | 1008 GB/s |
| Training epoch | 30 | 250 |
| Training time | ~3 min | ~3.5 h |
| Reconstruction | No reconstruction | ASP |
| Detection object | 99 nm-5.98 μm PS microspheres | 10 μm PS microspheres |
| Accuracy | > 98% | ~ 97% |

Table S4. *S. typhimurium* detection results in spiked pork samples using the UL-SNH biosensing platform (*n* = 3).

| **Spiked concentration (CFU/g)** | **Detected concentration（CFU/g）** | **Recovery rate (%)** | **Coefficient of variation (%)** |
| --- | --- | --- | --- |
| 1.0×10^3^ | 1.07×10^3^ | 107.3 | 12.7 |
| 1.0×10^4^ | 9.49×10^4^ | 94.9 | 8.2 |
| 1.0×10^5^ | 1.09×10^4^ | 109.3 | 7.5 |

Table S5. Comparison of UL-SNH, holography assay without nano-lens, and qPCR methods.

| **Method** | **UL-SNH (This work)** | **Holography assay without nano-lens (Previous work)** | **qPCR** |
| --- | --- | --- | --- |
| DNA extraction | Phage + RIPA lyse  (20 min) | Phage **+** RIPA lyse  (20 min) | Commercial DNA extraction kit (~ 4 h) |
| Capture efficiency | ~85% | ~80% | – |
| Temperature | 37℃ | 37℃ | 60–95℃, Cycle |
| Signal probe | 3 μm PS | 10 μm PS | FAM probes |
| Sensitivity | High | Middle | Normal |
| Cost | Low | Low | High |
| Time | ~1 h | **~**1.5 h | > 3 h |
| Operation | Easy (2 steps) | Middle (3 steps) | Difficult |
| Signal readout | Holography with nano-lens | Holography | Fluorescence |

Table S6**.** Performance comparison between UL-SNH and other methods for *Salmonella* detection.

| **Method** | **LOD (CFU/mL or /g)** | **Linear range (CFU/mL or /g)** | **Amplification** | **DNA extraction time (h)** | **Detection time (h)** | **Detection Cost** | **Viable identification** | **Reference** |
| --- | --- | --- | --- | --- | --- | --- | --- | --- |
| MARPS | 64 | 10^2^–10^7^ | PCR/RPA | ~ 4 | ~ 2 | Middle | No | ^[3]^ |
| Drop-CRISPR | 100 | 10^3^–10^7^ | PCR | ~ 4 | ~ 3 | Very high | No | ^[4]^ |
| CRISPR-MRS | 130 | 10^2^–10^6^ | PCR | ~ 4 | ~ 1.5 | High | No | ^[5]^ |
| RCA-CRISPR | 193 | 1.93×10^2^–1.93×10^8^ | RCA | ~ 4 | ~ 2 | High | No | ^[6]^ |
| QDs@MnO2 | 40 | 40–10^6^ | Amplification-free | – | ~ 2.25 | Middle | No | ^[7]^ |
| Pipette-adapted biosensor | 180 | 1.8×10^2^–1.8×10^6^ | Amplification-free | – | ~ 1.5 | Middle | No | ^[8]^ |
| GQDs-AuNPs-modified electrode | 356.2 | 10^3^–10^10^ | Amplification-free | – | ~ 4 | High | Yes | ^[9]^ |
| DPDPC | 72 | 10^2^–10^7^ | Amplification-free | ~ 0.3 | ~ 1.7 | Low | Yes | ^[2]^ |
| UL-SNH | 38 | 50–10^7^ | Amplification-free | ~ 0.3 | ~ 1 | Low | Yes | This work |

Table S7. Comparison between UL-SNH and other studies utilizing holography, CRISPR-based, or AI technology for biosensing.

| **Method** | **Imaging strategy** | **Imaging probe** | **Biosensing strategy** | **Image processing** | **Detection target** | **Reference** |
| --- | --- | --- | --- | --- | --- | --- |
| dCRISPR | Fluorescence microscopy | FQ–probe | CRISPR–Cas13a | ImageJ | SARS–CoV–2 | ^[10]^ |
| dWS–CRISPR | Fluorescence microscopy | FQ–probe | CRISPR–Cas12a | ImageJ | SARS–CoV–2 | ^[11]^ |
| CAT immunoassay | Bright-field microscope | 2–4 μm PS | Immunoassay | CV + ResNet  (*supervised*) | Proteins | ^[12]^ |
| FMDIA | Inverted microscope | r–QDMS | Immunoassay | ImageJ | Proteins | ^[13]^ |
| Microfluidic immunoassay platform | Dark-field microscope | AgNCs | Immunoassay | CNN  (*supervised*) | Cytokine | ^[14]^ |
| Particle cluster assay | Holography | PS bead cluster | Immunoassay | ASP + CNN (*supervised*) | HSV–1 | ^[15]^ |
| QLAB | Holography | 2 μm PS | Immunoassay | PSR + BP + ResNet (*supervised*) | SARS–CoV–2 | ^[16]^ |
| DPDPC | Holography | 10 μm PS | CRISPR–Cas12 | ASP+YOLOv7  (*supervised*) | Viable bacteria | ^[2]^ |
| UL-SNH | Holography with nano-lens | 99 nm–5.98 μm PS | Enhanced CRISPR–Cas12 | UL-CNN  (*Unsupervised*) | Viable bacteria | This work |

References

[1] Z. Wang, J. Liu, Y. Yang, P. Li, K. Li, Y. Xianyu, Y. Chen, B. Li, *Anal. Chem.* **2021**, *93*, 6178–6187.

[2] Y. Zhou, J. Zhao, R. Chen, P. Lu, W. Zhao, R. Ma, T. Xiao, Y. Dong, W. Zheng, X. Huang, B. Z. Tang, Y. Chen, *Nano Today* **2024**, *56*, 102238.

[3] Y. Lu, J. Wen, C. Wang, M. Wang, F. Jiang, L. Miao, M. Xu, Y. Li, X. Chen, Y. Chen, *Small* **2023**, *n/a*, 2308424.

[4] H. Wu, X. Cao, Y. Meng, D. Richards, J. Wu, Z. Ye, A. J. deMello, *Biosens. Bioelectron.* **2022**, *211*, 114377.

[5] Y. Shen, F. Jia, Y. He, Y. Fu, W. Fang, J. Wang, Y. Li, *Biosens. Bioelectron.* **2022**, *213*, 114437.

[6] X. Xiang, G. Xing, Y. Liu, Q. Wen, Y. Wei, J. Lu, Y. Chen, Y. Ji, S. Chen, T. Liu, Y. Shang, *J. Agric. Food Chem.* **2023**, *71*, 13518−13526.

[7] L. Xue, F. Huang, L. Hao, G. Cai, L. Zheng, Y. Li, J. Lin, *Food Chem.* **2020**, *322*, 126719.

[8] L. Wang, W. Qi, M. Wang, F. Jiang, Y. Ding, X. Xi, M. Liao, Y. Li, J. Lin, *Biosens. Bioelectron.* **2022**, *218*, 114765.

[9] N. Kumaragurubaran, P. Arul, S.-T. Huang, C.-H. Huang, S.-B. Fang, Y.-H. Lin, *Sens. Actuators, B* **2023**, *381*, 133428.

[10] D. Wang, X. Wang, F. Ye, J. Zou, J. Qu, X. Jiang, *ACS Nano* **2023**, *17*, 7250–7256.

[11] X. Ding, K. Yin, Z. Li, M. M. Sfeir, C. Liu, *Biosens. Bioelectron.* **2021**, *184*, 113218.

[12] W. Zhao, Y. Zhou, Y.-Z. Feng, X. Niu, Y. Zhao, J. Zhao, Y. Dong, M. Tan, Y. Xianyu, Y. Chen, *ACS Nano* **2023**, *17*, 13700–13714.

[13] F. Gong, Z. Tan, X. Shan, Y. Yang, S. Tian, F. Zhou, X. Ji, Z. He, *Anal. Chem.* **2024**, *96*, 3517–3524.

[14] Z. Gao, Y. Song, T. Y. Hsiao, J. He, C. Wang, J. Shen, A. MacLachlan, S. Dai, B. H. Singer, K. Kurabayashi, P. Chen, *ACS Nano* **2021**, *15*, 18023−18036.

[15] Y. Wu, A. Ray, Q. Wei, A. Feizi, X. Tong, E. Chen, Y. Luo, A. Ozcan, *ACS Photonics* **2019**, *6*, 294–301.

[16] C. J. Potter, Y. Hu, Z. Xiong, J. Wang, E. McLeod, *Lab Chip* **2022**, *22*, 3744–3754.
